# Photosensitizing deep-seated cancer cells with photoprotein-conjugated upconversion nanoparticles

**DOI:** 10.1186/s12951-023-02057-0

**Published:** 2023-08-19

**Authors:** Sung Hyun Park, Soohyun Han, Sangwoo Park, Hyung Shik Kim, Kyung-Min Kim, Suyeon Kim, Dong Yun Lee, Joonseok Lee, Young-Pil Kim

**Affiliations:** 1 Department of HY-KIST Bio-Convergence, Hanyang University, Seoul, 04763 Republic of Korea; 2 Department of Life Science, Hanyang University, Seoul, 04763 Republic of Korea; 3 Research Institute for Convergence of Basic Science, Hanyang University, Seoul, 04763 Republic of Korea; 4 Department of Bioengineering, College of Engineering, BK FOUR Biopharmaceutical Innovation Leader for Education and Research Group, Hanyang University, Seoul, 04763 Republic of Korea; 5 Department of Chemistry, Hanyang University, Seoul, 04763 Republic of Korea; 6 Institute of Nano Science and Technology, Hanyang University, Seoul, 04763 Republic of Korea; 7 Institute for Bioengineering and Biopharmaceutical Research, Hanyang University, Seoul, 04763 Republic of Korea; 8 Elixir Pharmatech Inc, Seoul, 04763 Republic of Korea; 9 Research Institute for Natural Sciences, Hanyang University, Seoul, 04763 Republic of Korea; 10 Hanyang Institute of Bioscience and Biotechnology, Hanyang University, Seoul, 04763 Republic of Korea

**Keywords:** Photodynamic therapy, Protein photosensitizers, Upconversion nanoparticles, Reactive oxygen species, Near-infrared light

## Abstract

To resolve the problem of target specificity and light transmission to deep-seated tissues in photodynamic therapy (PDT), we report a cancer cell-targeted photosensitizer using photoprotein-conjugated upconversion nanoparticles (UCNPs) with high target specificity and efficient light transmission to deep tissues. Core-shell UCNPs with low internal energy back transfer were conjugated with recombinant proteins that consists of a photosensitizer (KillerRed; KR) and a cancer cell-targeted lead peptide (LP). Under near infrared (NIR)-irradiating condition, the UCNP-KR-LP generated superoxide anion radicals as reactive oxygen species via NIR-to-green light conversion and exhibited excellent specificity to target cancer cells through receptor-mediated cell adhesion. Consequently, this photosensitizing process facilitated rapid cell death in cancer cell lines (MCF-7, MDA-MB-231, and U-87MG) overexpressing integrin beta 1 (ITGB1) receptors but not in a cell line (SK-BR-3) with reduced ITGB1 expression and a non-invasive normal breast cell line (MCF-10A). In contrast to green light irradiation, NIR light irradiation exhibited significant PDT efficacy in cancer cells located beneath porcine skin tissues up to a depth of 10 mm, as well as in vivo tumor xenograft mouse models. This finding suggests that the designed nanocomposite is useful for sensing and targeting various deep-seated tumors.

**Supplementary Information:**

The online version contains supplementary material available at 10.1186/s12951-023-02057-0.

## Background

Photodynamic therapy (PDT) has emerged as a promising treatment strategy for cancers and malignant diseases, which utilizes a light-activated photosensitizer (PS) in the presence of oxygen, inducing cell death by generating reactive oxygen species (ROS) [1–3]. However, despite the distinct advantages of minimal invasiveness and high spatiotemporal accuracy, clinical PDT applications have been limited to the treatment of superficial and endoscopically accessible lesions as surgical adjuvants. A main obstacle is insufficient light transmission to deep-seated tumors due to increased light absorption and scattering effects when visible light penetrates tissues [4]. To overcome this problem, lanthanide-doped upconversion nanoparticles (UCNPs) have been used in recent PDTs [5–11]. UCNPs are composed of a sensitizer and activator with different energy levels, which convert tissue-penetrating near-infrared (NIR) light into high-energy visible light with an efficiency orders of magnitude higher than that of multiphoton processes [12, 13]. In comparison with second-generation PSs including 5-aminolevulinic acid, hematoporphyrin derivatives, phenothiazine, chlorine, and phthalocyanine activated at wavelengths above 620 nm [14, 15], UCNPs in combination with green- or red-emitted PSs respond to much longer wavelengths of up to 1,100 nm, making them more effective in accessing deep tissues within the light transmission range for biological applications (700−1,100 nm) [16]. However, two challenging issues remain when using conventional PSs for UCNP-based PDT: high hydrophobicity and low target specificity. PS-conjugated nanoparticles could form aggregates in body fluids under a physiological milieu due to self-aggregation or insolubility [17, 18]. Importantly, most of them rely on passive targeting via leaky endothelial cell layers in tumor vascular tissues, which generally requires high-dose nanoparticles and long-term light irradiation for tumor destruction. Although some tumor-targeting ligands have been attached onto the surface of UCNPs for use in PDT [5, 6, 11, 19], the nanocomposites including chemical PSs require extensive synthetic modifications, and pharmacokinetic properties are difficult to control, thus deteriorating the efficiency and accuracy of repeated PDTs. Alternative to chemical PSs, light-sensitive proteins with controlled expression and biodegradability have been considered as effector molecules for optogenetic ROS production [20]. Therefore, we reason that UCNP-modified ROS-generating proteins with targeting capability would expand the usefulness of PDT.

We report the use of photoprotein-bound UCNPs for the detection and treatment of deep-seated cancer cells to resolve the problem of specificity and solubility simultaneously. The recombinant photoprotein consists of a ROS-generating photoprotein and a tumor-targeting lead peptide (LP). We adopted a dimeric KillerRed (KR) as the proteinaceous PS, which is a genetically engineered red fluorescent protein variant that can generate ROS and fluorescence at an excitation wavelength of ~ 550 nm [21]. As a peptide ligand for targeting cell surface receptors, the LP comprising 10 amino acid sequences (WLEAAYQRFL) was linked to the C-terminus of KR. We reported in a previous study that LP could bind to integrin beta 1 (ITGB1) receptors overexpressed in various cancer cells [22]. In the nanocomposite (UCNP-KR-LP) created by crosslinking the recombinant KR-LP to the UCNP, KR-LP allows target specificity and photoactive ROS generation, whereas the UCNP functions as both a nanocarrier of KR-LP and a transducer that transfers light energy to KR-LP in response to NIR (~ 980 nm). In comparison with classical co-doped (Yb^3+^/Er^3+^) UCNPs, the separation of the Yb^3+^ sensitizer and Er^3+^ activator in NaYF_4_:Yb^3+^@NaYF_4_:Er^3+^ (sensitizing core@activating shell) UCNPs can avoid energy back transfer (EBT) from the activator to the sensitizer in the UCNPs and reduce the distance between the donor (activator in the UCNPs) and the acceptor (KR). Therefore, the efficiency of NIR-to-visible internal energy transfer (IET) as well as fluorescence resonance energy transfer (FRET) efficiency from the donor to the acceptor can be improved.

In terms of target specificity without affecting normal healthy cells/tissues, we observed previously that KR with a peptide ligand had a stronger lethal effect on cancer cells compared with that of KR without a peptide ligand, which may be attributed to a difference in receptor-mediated interactions between KR and cancer cells [22]. Indeed, active cellular targeting strategies with affinity ligands instead of passive targeting can increase retention at the target site as well as uptake by the target cells via an enhanced permeability and retention (EPR) effect; only a small proportion of nanocarriers (less than 1%) in passive targeting can be delivered to the target site even in high-EPR xenografted tumors [23]. For this reason, we examined the receptor-mediated and active targeting effect of UCNP-KR-LP on the growth of 5 cancer cell lines (MCF-7, SK-BR-3, MDA-MB-231, MCF-10 A, and U-87MG) under NIR irradiation through cytotoxic assay and confocal fluorescence imaging. This approach is different from a previous method as it directly conjugates KR with UCNPs as an agent for passive targeting in PDT [24]. Moreover, to demonstrate the tissue permeability of our PDT agent, we compared the effectiveness of NIR- and visible light-sensitizing PDT using UCNP-KR-LP between porcine skin tissues with different thicknesses (0−10 mm). Given the short lifespan (from ns to µs) and narrow diffusion distance (< 50 nm) of PS-generated singlet oxygen (^1^O_2_) or superoxide (O_2_^−•^) under physiological conditions [25–27], we hypothesized that despite the usability of targeted antibodies, the peptide ligands of UCNPs could contribute to high stability and allow the near-field delivery of specific ROS to tumor cells without the need for complex surface modifications.

## Materials

Yttrium (III) acetate hydrate (99.9%), ytterbium (III) acetate hydrate (99.9%), erbium (III) acetate hydrate (99.95%), oleic acid (OA, 90%), 1-octadecene (ODE, 90%), sodium hydroxide (NaOH, > 98%), ammonium fluoride (NH_4_F, 99.9%), methanol (99.8%), ethanol (absolute), cyclohexane (99%), tetrahydrofuran (THF, 99.9%), dopamine hydrochloride (99.9%), hydrochloric acid (HCl, 37%), dimethyl sulfoxide (DMSO, 99.9%), hydroxylamine hydrochloride (HH, 99%), superoxide dismutase *(*SOD), sodium azide (NaN_3_), and D-mannitol were purchased from Sigma-Aldrich (St Louis, MO, USA). HEPES buffer (1 M, pH 7.2−7.5), N-succinimidyl S-acetylthioacetate (SATA), sulfo-SMCC (sulfosuccinimidyl 4-(N-maleimidomethyl) cyclohexane-1-carboxylate), dihydroethidium (DHE), and SYTOX Green (SG) were purchased from Thermo Fisher Scientific (Waltham, MA, USA). Amicon™ Ultra Centrifugal Filter (0.5 mL, 30 K, 5 K) was purchased from Millipore (Bedford, MA, USA). 2ʹ,7ʹ-Dichlorofluorescein diacetate (DCFDA) was purchased from Cell Biolabs (San Diego, CA, USA). 4′,6-Diamidino-2-phenylindole (DAPI) was obtained from Vector Laboratories (Newark, CA, USA). 1,4-Dithiothreitol (DTT) was purchased from Duchefa Biochemie (RV Haarlem, Netherlands).

## Methods

### Characterization

The morphologies of the UCNPs were characterized by transmission electron microscopy using JEM-2100 F (JEOL Ltd., Tokyo, Japan) installed at Hanyang LINC3.0 Analytical Equipment Center (Hanyang University, Seoul, Republic of Korea) at an accelerating voltage of 200 kV. The XRD patterns of the UCNPs were analyzed by grazing incidence X-ray diffraction (GI-XRD) with a fixed incidence angle of 1 °, a measuring range from 10 ° to 60 ° for a 0.04 ° step, and a scan speed of 2 ° per min. A high-resolution X-ray diffractometer (Smartlab, Rigaku) with a HyPix-3000 detector and Cu-K (= 1.54) radiation operating at 9 kW was used for GI-XRD. DLS and zeta potential measurements of the UCNPs were performed using a Zetasizer Nano ZSP instrument (Malvern Co., Malvern, UK). Fourier transmission infrared (FT-IR) spectra of the UCNPs were obtained using an iS50 FTIR spectrophotometer (Thermo Fisher Scientific). The upconversion PL emission spectra were recorded by the sCMOS camera (Andor, ISTAR-SCMOS-18 F-73) attached to Andor’s Kymera 193i spectrometer with external laser excitation at 980 nm (Changchun New Industries Optoelectronics Tech. Co. Ltd. (CNI), Jilin, China). The emission at 610 nm was measured using bandpass filters (ff-01-800/12–25; Edmund Optics, Barrington, NJ, USA) placed in front of the spectrometer. The PL lifetime was measured by exciting the samples with a pulsed 980 nm laser (MDL-III-980; CNI) at a repetition rate of 100 Hz and a pulse width of 100 µs. The emission at 550 nm was selectively measured using a photomultiplier tube detector (H10721-01; Hamamatsu, Shizuoka, Japan) attached to Andor’s Kymera 193i spectrometer, the signal of which was acquired using a digital oscilloscope (RTM3002; Rhode & Schwarz, Munich, Germany). A 550 nm laser (MGL-FN-550; CNI) was used to measure the transmittance of green light to deep tissues.

### Preparation of core precursor solution

We prepared the lanthanide precursor solution in a solvent mixture of OA and ODE. In a typical process of preparing the core precursor, Ln(CH_3_CO_2_)_3_ (Ln = Y, Yb, Er total 0.8 mmol) was mixed with 4 mL of OA and 6 mL of ODE. The mixture was heated at 153 °C for 1 h with magnetic stirring and then cooled down to room temperature. Subsequently, a 5 mL methanol solution of NaOH (0.25 mmol) and NH_4_F (0.4 mmol) was added to the oleate-lanthanide solution. The reaction mixture was stirred at 50 °C for 1 h. Then, the solution temperature was increased to 110 °C, followed by degassing through a vacuum pump at 103 °C for 10 min to remove methanol. Finally, the precursor solution was cooled to room temperature and stored in a 50 mL centrifugal tube.

### Synthesis of core nanoparticles

In a typical process of core UCNP synthesis, a mixture of 4 mL of OA and 6 mL of ODE was loaded into a 50 mL round bottom flask. Then, the mixture was heated to 103 °C for 30 min. Subsequently, the solution was heated to 310 °C under argon protection. After the solution temperature reached 310 °C, 5 mL of the core precursor was rapidly injected with a one-shot approach, and the solution was stirred at 310 °C for 60 min. The resulting nanoparticles were collected by centrifugation, washed with ethanol, and dispersed in cyclohexane.

### Synthesis of β-NaYF_4_:x% Yb^3+^@NaYF_4_:2% Er^3+^ (x = 20, 30, 40, 50 mol%) core-shell nanoparticles

For the synthesis of UCNPs constituted with β-NaYF_4_:x% Yb^3+^@NaYF_4_:2% Er^3+^(*x* = 20, 30, 40, 50 mol%), Ln(CH_3_CO_2_)_3_ (Ln = Y, Er, total 0.2 mmol) was mixed with 3 mL of OA and 7 mL of ODE. The mixture was heated at 153 °C for 1 h with magnetic stirring and then cooled down to room temperature. Subsequently, the pre-synthesized core nanoparticles were added as seeds with a 3 mL methanol solution of NaOH (0.25 mmol) and NH_4_F (0.4 mmol). The reaction mixture was stirred at 50 °C for 1 h and heated at 300 °C under an argon flow for 1 h before cooling to room temperature. The resulting core-shell nanoparticles were collected by centrifugation, washed with ethanol, and dispersed in cyclohexane.

### Surface modification of UCNPs

Dopamine hydrochloride aqueous solution (200 µL, 25 wt %) and UCNPs were dissolved in THF. The solution was added to a flask and heated to 50 °C with vigorous stirring. After adding HCl, the resulting UCNP-NH_2_ was collected through several washing steps. To prepare maleimide-modified UCNPs, 0.5 mg of UCNP-NH_2_ was resuspended in 10 mM HEPES buffer, and sulfo-SMCC was dissolved in 200 µL of 10 mM HEPES buffer. The UCNP-NH_2_ and SMCC solutions were mixed and incubated for 5 h. After incubation, the resulting UCNP-SMCC solution was washed. To prepare thiol-modified KR-LP, SATA stock solution was added to KR-LP (50 mg), which was incubated for 30 min. HH stock solution (2 µL at 0.5 M) was added to the reaction solution and incubated for 2 h. Finally, the prepared thiol-modified KR-LP and maleimide-modified UCNPs were mixed in HEPES and incubated for 2 h. The thiol group-modified KR-LP was reacted with the maleimide groups on the UCNP surface at pH 7.2. The KR-LP-conjugated UCNP solution was collected through centrifugation. To determine the KR-LP loading amount, the supernatant and residual content were measured using a Nanodrop spectrophotometer at a wavelength of 280 nm.

## Expression and refinement of recombinant proteins

For cancer cell targeting, the KR-LP recombinant protein was produced by introducing the LP sequence (Trp–Leu–Glu–Ala–Tyr–Gln–Arg–Phe–Leu) into the pRSET_B_–His_6_–KR vector at the C-terminal of KR. This was amplified using an omnidirectional primer (forward primer, 5’–GGG GAT CCC ATG CTG TGC TGT ATG AGA A–3’) and three reverse primers (reverse primers, 5’–CCA ATC CTC GTC GCT ACC GAT GGC G–3’, 5’–CTG GTA GGC GGC CTC CAG CCA ATC–3’, 5’–CCC AAG CTT CTA CAG GAA GCG CTG GTA G–3’). PCR products were cut using restriction enzymes (*BamH*I and *Hind*III), separated by agarose gel electrophoresis, and purified using a gel extraction kit. Ligation to the vector was performed at 16 °C for 2 h using T4 DNA ligation enzyme. Subsequently, the recombinant vector was transformed into *E.coli* DH5α strain to extract DNA and transformed into *E.coli* BL21 (DE3) strain (Novagen, Madison, WI, USA) for protein expression. The transformed *E.coli* BL21 (DE3) strain was inoculated into 100 mL of Luria broth (LB) culture medium containing ampicillin at a concentration of 100 mg/mL and incubated at 37 °C for 24 h for the expression and purification of recombinant proteins. Then, it was inoculated into 500 mL of LB culture solution and cultured at 37 °C until the absorbance (optical density, OD) value reached 0.6–0.8. After leaving it at 4 °C for 30 min to reduce the temperature of the culture solution, isopropyl β-D-1-thiogalactopyranoside (IPTG) was added to reach a final concentration of 1 mM, followed by incubation at 20 °C for 20 h to induce protein expression. The cells were centrifuged at 7,800 rpm at 4 °C for 20 min and resuspended with 40 mL of lysis buffer (50 mM NaH_2_PO_4_, 300 mM NaCl, 10 mM imidazole, pH 8.0), followed by sonication for 5 min. Then, the cells were centrifuged at 7,800 rpm at 4 °C for 20 min, and the supernatant solution was filtered through a 0.45 μm syringe filter. Subsequently, the Ni^2+^-NTA resin was added, and the solution was incubated for 24 h at 4 °C. The protein and bead mixture solution was loaded into a polypropylene column, washed 3 times with 5 mL of washing buffer (50 mM NaH_2_PO_4_, 300 mM NaCl, 20 mM imidazole, pH 8.0), and purified with 2.5 mL of elution buffer (50 mM NaH_2_PO_4_, 300 mM NaCl, 250 mM imidazole, pH 8.0). The size and fluorescence of the purified protein were confirmed by 12% SDS gel electrophoresis. Then, the protein purified with the eluted solution was desalted with PD-10 columns and concentrated using a 50 kDa Amicon Ultra Centrifugation Filter. The protein concentration (mg/mL) was determined by measuring the absorbance at a wavelength of 280 nm using a UV-vis instrument (Cary 60; Agilent Technology), and the sample was stored at − 80 °C for further experiments.

### Preparation of UCNP-KR-LP

To produce UCNP-maleimide, 0.5 mg of UCNP-NH_2_ was resuspended in 10 mM HEPES buffer, and sulfo-SMCC was dissolved in 200 µL of 10 mM HEPES buffer. Then, the UCNP-NH_2_ solution and SMCC solution were mixed and reacted for more than 5 h. The SMCC-UCNP solution was washed with 10 mM HEPES buffer. Next, thiol-KR-LP was prepared by adding a SATA-diluted solution to KR-LP, reacting it for 30 min, and incubating the 0.5 M HH-diluted solution for 2 h. Next, the prepared thiol-KR-LP was mixed with UCNP-maleimide and HEPES buffer and reacted for 2 h. Subsequently, it was washed 3 times by centrifugation to remove unreacted material. Finally, the synthesized UCNP-KR-LP composite was stored at 4 °C at a final volume of 200 µL. Based on the extinction coefficient value of dimeric KR-LP at 580 nm and the relative extinction of CS-UCNP with a core diameter of 20 nm at the same wavelength, the estimated number of KR-LP per CS-UCNP in the conjugate was approximately 37 ± 2.

### Cell culture and MTT assay

MCF-7, MDA-MB-231, U87-MG, SK-BR-3, and MCF-10 A cells were cultured in a T-75 cell culture plate using a cell culture medium containing 10% FBS, and 1% penicillin-streptomycin (5% CO_2_, 37 °C). The MCF-7, MDA-MB-231, and U87-MG cancer cell lines were cultured using DMEM, the SK-BR-3 cancer cell line was cultured using RPMI medium, and the MCF-10 A cell line (non-malignant) was cultured using mammary epithelial basal medium. At 70% confluency, the cells were washed thrice with DPBS and collected by centrifugation after treatment with 3 mL of trypsin-EDTA. Subsequently, trypan blue was added, and the number of cells was counted using a hematocytometer. Next, the cells were cultured in a 96-well plate at a concentration of 5 × 10^3^ cells per well and incubated for 24 h at 37 °C. UCNP, UCNP-KR, and UCNP-KR-LP were added at a final concentration of 100 µg/mL and incubated for 16 h. After removing the culture medium, cells were washed thrice with DPBS and irradiated with a 980 nm NIR laser (1 W/cm^2^) for 30 min. Next, the cells were washed twice with DPBS, and MTT reagent (0.5 mg/mL) was added and incubated for 4 h at 37 °C. After removing the supernatant, 100 µL of DMSO was added to the medium and stored for another 10 min. Subsequently, the absorbance was measured using a microplate reader (Vashokan, Thermo Scientific, USA) at 570 nm with a colorimetric indicator (formazan).

### ROS measurement

DHE was used to measure the level of superoxide (O_2_^•–^) generated by KR. The final concentration of the UCNP-KR-LP composite prepared by mixing the DHE reaction buffer with PBS was 100 µg/mL, and the final concentration of DHE was 100 µM. NIR irradiation was examined at 10 min intervals using a 980 nm laser for a total of 30 min, and the amount of DHE fluorescence reduction was determined at an excitation wavelength of 370 nm and an emission wavelength of 420 nm using a microplate reader. To confirm the scavenging effect of ROS generation, we used SOD (superoxide anion-specific scavenger), NaN_3_ (singlet oxygen-specific scavenger), and D-mannitol (C_6_H_14_O_6,_ hydroxyl radical specific scavenger). The experiment was conducted by mixing the three scavengers with KR in the ROS reaction buffer. The final concentration was 100 µg/mL KR and 100 µM DHE. Subsequently, the amount of DHE fluorescence reduction before and after light irradiation was measured using a microplate reader. A fluorogenic ROS probe, DCFDA, was used to measure intracellular ROS production. MCF-7 cells were cultured in a 96-well culture plate (5 × 10^3^ cells per well) and incubated for 24 h at 37 °C. Then, UCNP, UCNP-KR, and UCNP-KR-LP were added at 100 µg/mL for 16 h. After removing the culture medium, 10 µM DCFDA was added and cultured in the dark. Cells were washed twice with DPBS and irradiated with a 980 nm NIR laser (1 W/cm^2^) for 30 min. After NIR irradiation, the DCFDA fluorescence signals of cells were detected at an excitation wavelength of 480 nm and an emission wavelength of 530 nm using a flow cytometer (FACSCanto II; BD Biosciences, Franklin Lakes, NJ, USA), microplate reader, and fluorescence imaging system (EVOS M7000 Imaging System, Thermo Fisher Scientific). Flow cytometry was performed by treating the cells with 50 µL of trypsin-EDTA for 1 min and removing the medium from the 96-well culture plate. Then, the plate was washed twice with DPBS, and the cells were filtered with a cell strainer and placed in a cap tube. The cells were kept in an ice bath during analysis, and fluorescence signals were analyzed by detecting DCFDA in the fluorescein (FITC) channel. The experimental results were analyzed through FlowJo (FlowJo™ v10.7; BD Biosciences).

### Fluorescence imaging of cells

MCF-7 cells were cultured in a 24-well plate as described above, treated with 200 µg/mL UCNP-KR or UCNP-KR-LP for 16 h, and irradiated with a NIR laser (1 W/cm^2^) for 30 min. SYTOX Green (a dye that stains the nucleus of dead cells in green) was then added to a final concentration of 261 nM with DAPI (a dye for staining the nucleus of live cell in blue), and fluorescence images were obtained using a confocal fluorescence microscope (Eclipse Ti; Nikon). Experiments on the transmittance of green and NIR light to deep tissues were conducted by placing porcine skin tissues of various thicknesses (0−10 mm) in a 24-well culture plate with MCF-7 cells under a 550 nm green laser and a 980 nm NIR laser for 30 min. To obtain tissues with an accurate thickness, the slice thickness of the tissues was measured using a caliper. The rest of the experimental procedure is similar to the method described above.

### In vivo experiment

All animals were maintained under specific pathogen-free conditions and in strict accordance with the guidelines established by the Institutional Animal Care and Use Committee (IACUC: 2020–0081) at Hanyang University. Adhering to the animal ethics guidelines outlined by the IACUC at Hanyang University, BALB/c nude mice (*n* = 3; Nara-Bio Company, Seoul, Korea) were employed to establish tumor-bearing mouse model. The administration was achieved by subcutaneously injecting 1 × 10^6^ MDA-MB-231 cells mixed with Matrigel (Corning, NY, USA) into both flanks of the nude mice. Once the tumor volumes reached approximately 100 mm^3^ after 9 days, the mice were randomly assigned to receive treatment. Direct injections of a 100 µL solution of UCNP-KR (200 µg/mL in PBS) and UCNP-KR-LP (200 µg/mL in PBS) were administered into the left and right tumor sites on the dorsal side of the mouse, respectively. Two hours after injecting the nanocomposite, a 980 nm laser irradiation was conducted at a power density of 1.5 W/cm^2^ for 10 min, implementing 1-min on/off intervals to prevent water overheating. These nanocomposite injections and subsequent NIR irradiations were repeated three times on days 9, 11, and 13, and the tumor volume was measured daily until day 21.

### Histological analysis of tumor tissue

The subcutaneously established tumor tissues were fixed in 4% paraformaldehyde and processed automatically using the Leica TP1020 semi-enclosed Benchtop Tissue Processor (Hesse, Germany). Following embedding in paraffin, the paraffin blocks were sliced into 7 μm thick sections using the Leica RM 2145 Microtome. For histological analysis, the prepared sections were stained with hematoxylin and eosin to visualize the cellular structures.

### Inductively coupled plasma mass spectrometry (ICP-MS)

To quantitatively measure the elemental content of UCNPs by cellular uptake, we employed ICP-MS (iCAP RQ, Thermo Fisher Scientific) analysis. MDA-MB-231 cells were cultured in a 6-well plate until they reached 80% confluency. Each nanocomposite solution (CS-UCNP, CS-UCNP-KR, or CS-UCNP-KR-LP) was prepared in DMEM cell culture media supplemented with 10% FBS and 1% P/S. Prior to the UCNP treatment, the cells underwent thorough washing with PBS. Subsequently, 2 mL of DMEM media containing each nanocomposite at a concentration of 200 µg/mL was added to the cell plate. The MDA-MB-231 cells were incubated at 37 ℃ for 20 min. After the incubation period, unbound UCNP composites were removed by washing the cells with PBS buffer. The UCNP composite taken up by the cells were subsequently collected through centrifugation, and the presence of yttrium ions was analyzed using ICP-MS.

### Statistical analysis

Statistical analysis was performed using the SPSS software (version 26.0; IBM). Statistical significance was determined by *t*-test (for two groups) or one-way ANOVA with post-hoc Tukey’s test (for more than three groups) as indicated in the figure legends. All data sets were subjected to normality tests (Shapiro-Wilk test) when applicable. Based on the normality of distributions, *t*-test or one-way ANOVA with post-hoc Tukey’s test was performed when the data set was truly normal. *N* values corresponded to the sample size, and *P*-values were as follows: *P < 0.05; **P < 0.01; ***P < 0.001.

## Results and discussion

### Construction and characterization of UCNP and UCNP-KR-LP

**Fig. 1:**
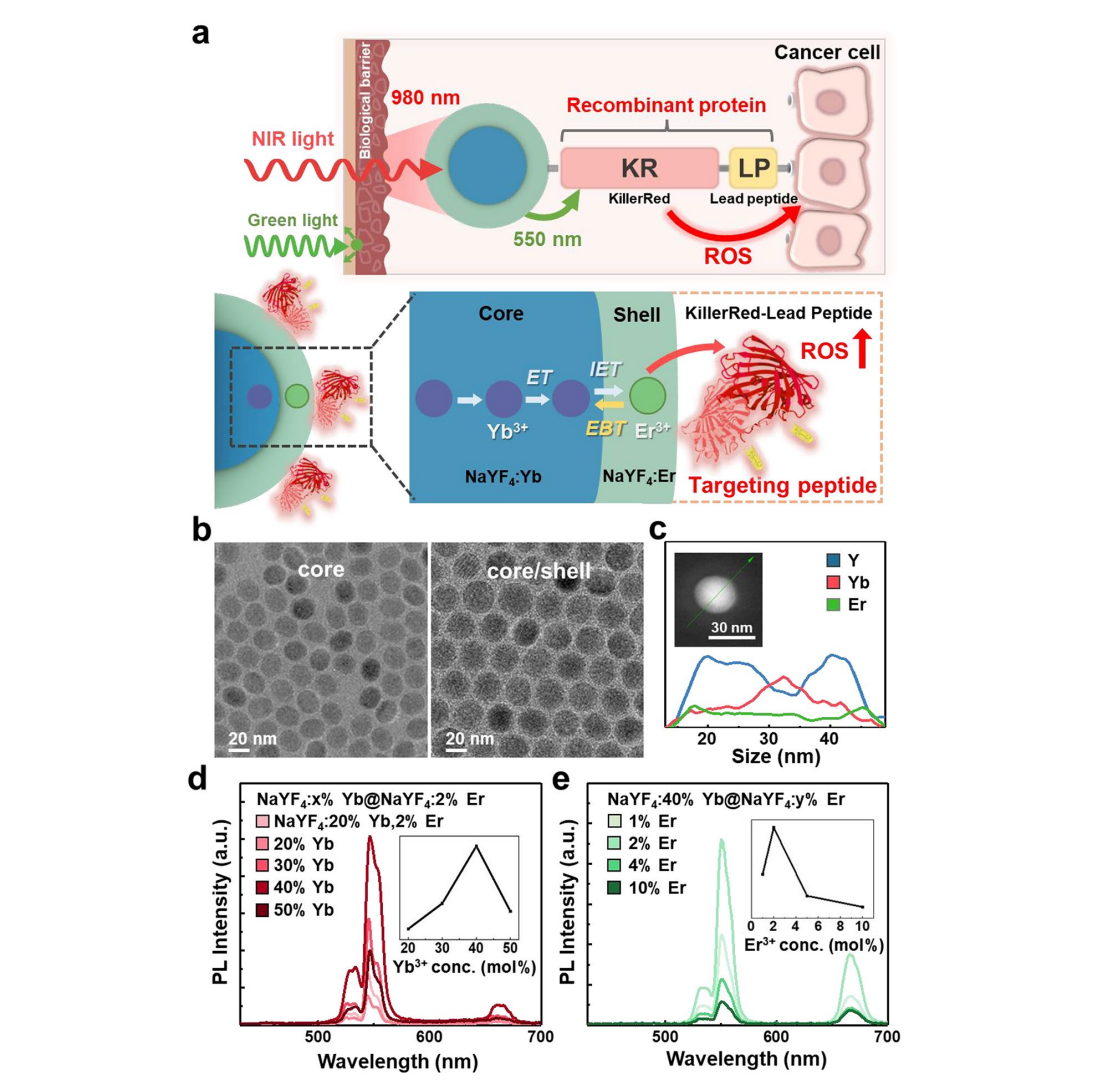
Schematic and construction of CS-UCNPs and Co-UCNPs as cancer cell-targeted PDT agents. **a** Schematic illustrations of cancer cell-targeted PDT using UCNP-KR-LP (top) and an efficient photon ET pathway from UCNPs to KR (bottom). **b** TEM images of core (NaYF_4_:Yb^3+^) and core-shell (NaYF_4_:Yb^3+^@NaYF_4_:Er^3+^) UCNPs. **c** EDS spectrum of CS-UCNPs (NaYF_4_:Yb^3+^@NaYF_4_:Er^3+^). The inset shows EDS line scan analysis of the TEM image of a single CS-UCNP. **d−e** PL intensity spectra of core (NaYF_4_:*x*% Yb^3+^, 2% Er^3+^) and core-shell (NaYF_4_:40% Yb^3+^@NaYF_4_:*y*%Er^3+^) UCNPs at an excitation wavelenth of 980 nm with varying Yb^3+^ concentrations in the core (**d**) and Er^3+^ doping concentrations in the outer layer (**e**). The inset shows the PL intensity at 550 nm as a function of the concentration (mol%) of either Yb^3+^ or Er^3+^. CS-UCNP, core-shell upconversion nanoparticle; Co-UCNP, co-doped upconversion nanoparticle; KR, KillerRed; LP, lead peptide; ROS, reactive oxygen species; PL, photoluminescence

As illustrated in Fig. 1a, we designed UCNP-KR-LP as a PDT agent by conjugating core-shell UCNPs (termed CS-UCNPs hereafter) with recombinant photoproteins (KR-LP) via chemical crosslinking. The synthesized CS-UCNPs were composed of a sensitizing core and an activating shell (NaYF_4_:Yb^3+^@NaYF_4_:Er^3+^) to efficiently convert NIR light at 980 nm into visible green light at 550 nm, which can be transmitted to KR-LP via energy transfer (ET). CS-UCNP functioned as a nanocarrier and NIR transducer, and KR-LP functioned as a ROS-generating proteinaceous PS (for KR) and tumor-targeting moiety (for LP). Efficient IET from the NaYF_4_:Yb^3+^ core to the NaYF_4_:Er^3+^ shell and reduced EBT in UCNPs could induce ROS-mediated cell death through strong interactions between the LP and receptors in cancer cells. The LP ligand (WLEAAYQRFL) is a sequence known to have high binding affinity for breast cancer cells and neuroblastoma cells, as demonstrated previously by phage display or peptide microarray [28, 29].

Based on transmittance electron microscopy (TEM) images of the primitive core and constructed CS-UCNPs, the NaYF_4_:Yb^3+^ core had a spherical shape with an average diameter of 18.2 ± 1.1 nm, and the NaYF_4_:Er^3+^ shell surrounding the NaYF_4_:Yb^3+^ core had larger diameter of 27.1 ± 1.1 nm (Fig. 1b). Following line scan analysis of a single core-shell in energy-dispersive X-ray spectroscopy (EDS), the spectrum of CS-UCNPs was obtained, which showed the elemental characteristics of dopants; Yb^3+^ ions were located in the core area, whereas Er^3+^ ions covered the core particles and were concentrated in the shell area (Fig. 1c). In addition, the presence of template ions (F^−^, Na^+^, and Y^3+^) and rare-earth dopant ions (Yb^3+^ and Er^3+^) was detected by EDS mapping (Additional file 1: Figure S1), and the X-ray diffraction (XRD) patterns of CS-UCNPs revealed standard diffraction peaks of the hexagonal NaYF_4_ structure (JCPDS no. 28-1182) (Additional file 1: Figure S2). These results demonstrated the distinct core-shell structure of the synthesized CS-UCNPs with the spatial separation of Yb^3+^ and Er^3+^ ions in each core and shell layer. In addition, we optimized the photoluminescence (PL) emission spectra of CS-UCNPs by adjusting the concentrations of sensitizer and activator ions. When we varied the concentration of the NaYF_4_:*x*% Yb^3+^ core (*x* = 20, 30, 40, 50 mol%) with a fixed concentration of the NaYF_4_:2% Er^3+^ shell, the PL intensity of NaYF_4_:40% Yb^3+^@NaYF_4_:2% Er^3+^ CS-UCNPs at an emission wavelength of 550 nm under 980 nm irradiation (1 W/cm^2^) was 5.4 times higher compared with that of conventional NaYF_4_:20% Yb^3+^, 2% Er^3+^ co-doped UCNPs (Co-UCNPS) (Fig. 1d). The PL intensity of CS-UCNPs reached the maximum at 2% of the Er^3+^ shell with a fixed concentration of the NaYF_4_: 40% Yb^3+^ core; an Er^3+^ concentration of more than 2% markedly decreased the PL intensity (Fig. 1e). Under various concentrations of Yb^3+^or Er^3+^, the particle diameters of conventional Co-UCNPs (NaYF_4_:x% Yb^3+^, 2% Er^3+^) and CS-UCNPs (NaYF_4_:*x*% Yb^3+^@NaYF_4_:2% Er^3+^ or NaYF_4_:40% Yb^3+^@NaYF_4_:*y*% Er^3+^; *y* = 1, 2, 5, 10 mol%) were maintained at ~ 18 nm and ~ 27 nm, respectively (Additional file 1: Figure S3−S5). Unlike CS-UCNPs, Co-UCNPs (NaYF4:*x*% Yb^3+^, 2% Er^3+^; *x* = 20, 30, 40, 50 mol%) exhibited a decrease in PL intensity at high Yb^3+^ ion doping levels due to the increased Yb^3+^-Er^3+^ cross-relaxation (reverse ET) (Additional file 1: Figure S6a). This observation indicates superior stability of the core-shell structure, even at high concentrations of Yb^3+^ ions compared to the core structure. Importantly, the lifetime of CS-UCNPs was much longer than that of Co-UCNPs when the Er^3+^ emission decay was measured at 550 nm under the same Er^3+^ composition (Additional file 1: Figure S6b). These results indicated that the spatial separation of the sensitizer (Yb^3+^) and activator (Er^3+^) in CS-UCNPs into different layers may be responsible for enhancing PL emission with efficient IET, thus demonstrating that the core-shell structure may efficiently suppress EBT at a high Yb^3+^ ion doping level, as reported previously [30, 31].

**Fig. 2:**
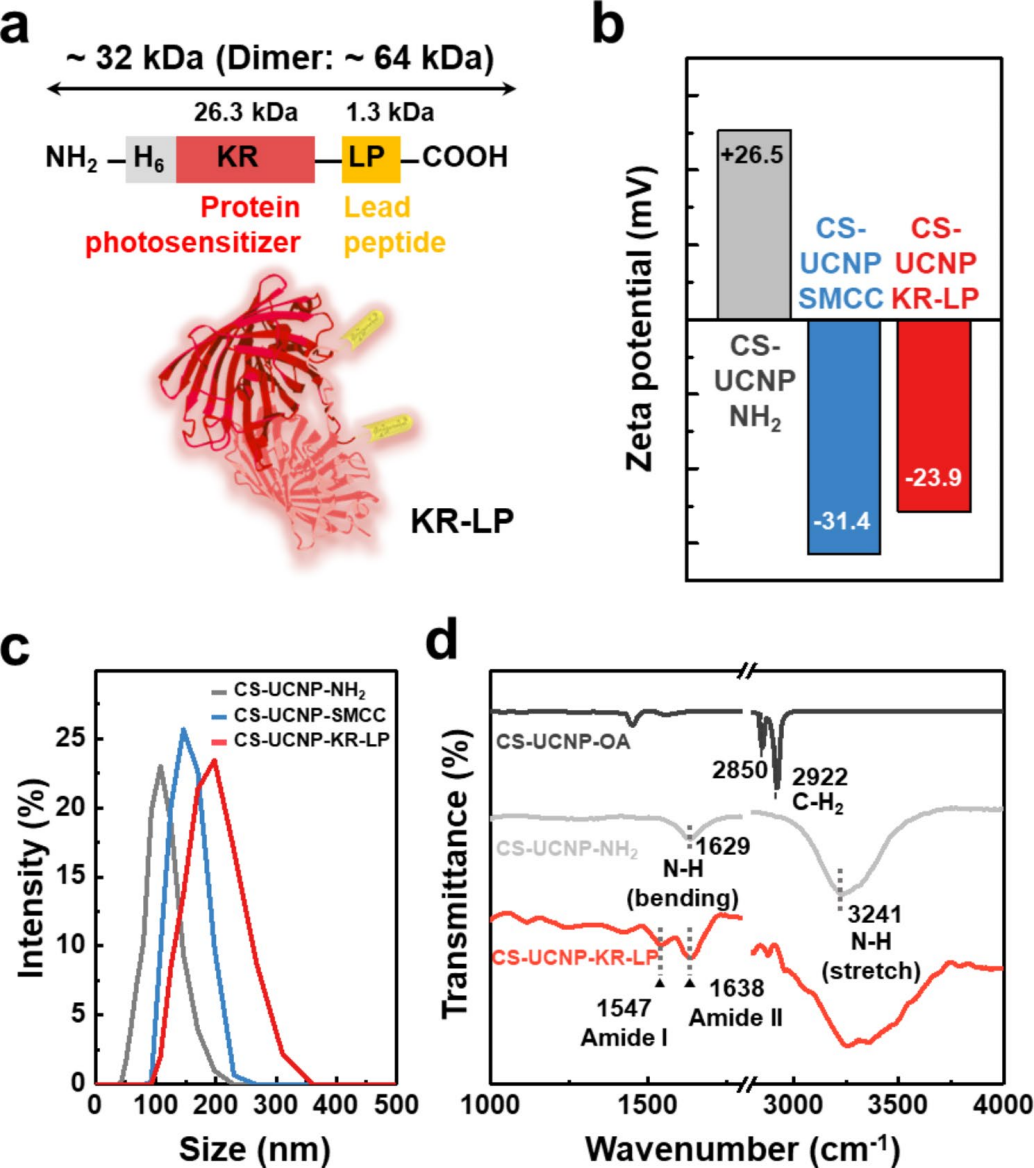
Construction and characterization of UCNP-KR-LP. **a** Schematic of the primary structure of a recombinant protein (KR-LP) from the N to C-terminus. **b−c** Zeta-potential values (**b**) and DLS hydrodynamic particle size distributions (**c**) of CS-UCNP-NH_2_, CS-UCNP-SMCC, and CS-UCNP-KR-LP. **d** FT-IR spectra of CS-UCNP-OA, CS-UCNP-NH_2_, and CS-UCNP-KR-LP. DLS, dynamic light scattering; FT-IR, Fourier transform infrared spectroscopy

For the construction of UCNP-KR-LP, the recombinant protein (KR-LP) was chemically conjugated on the surface of NaYF_4_:40% Yb^3+^@NaYF_4_:2% Er^3+^ CS-UCNPs, where amine-functionalized CS-UCNPs were treated with thiolated KR-LP using a hetero-bifunctional crosslinker (sulfo-SMCC, containing N-hydroxysuccinimide (NHS) ester and maleimide groups). KR-LP was expressed as an inherent homodimeric protein (~ 64 kDa) as KR is derived from a *Hydrozoa*-derived red fluorescent protein maturated by the obligate dimerization process [32, 33]. Although the two N-termini of the dimeric KR β-barrels are more separated (~ 60 Å) than the two C-termini (~ 44 Å) [34], the LP was fused to the adjacent C-termini of KR, allowing bivalent binding without the structural constraints of KR (Fig. 2a). The dimeric sizes of KR and KR-LP were verified by sodium dodecyl sulfate polyacrylamide gel electrophoresis (SDS-PAGE) and fluorescent gel imaging (Additional file 1: Figure S7). The surface charges of CS-UCNP-SMCC (NHS/maleimide modification) and CS-UCNP-KR-LP (protein modification) were changed to negative values (−31.4 ± 1.1 mV and −23.9 ± 0.5 mV, respectively) in contrast to the charge (+ 26.5 ± 0.8 mV) of unmodified CS-UCNP-NH_2_ (Fig. 2b), which may be primarily attributed to the modification of primary amines and the isoelectric point (p*I* ≈ 5.4) of KR. The hydrodynamic size of CS-UCNP-KR-LP measured by dynamic light scattering (DLS) was also increased following the modification of CS-UCNP-NH_2_ (Fig. 2c). When CS-UCNP-OA was converted into CS-UCNP-NH_2_, Fourier transform infrared spectroscopy (FT-IR) spectra showed increased peak intensities at 3,241 and 1,629 cm^− 1^ (− NH_2_ group) with a significant decrease in peak intensities at 2,922 and 2,850 cm^− 1^ (− CH_2_ group) (Fig. 2d). Notably, distinct amide bands at 1,638 cm^− 1^ (amine II), and 1,547 cm^–1^ (amide I) were observed in CS-UCNP-KR-LP, consistent with the characteristic peaks of KR-LP (Additional file 1: Figure S8). These results strongly indicated the presence of KR-LP on the surface of CS-UCNPs. In addition, when we assessed in vitro stability of CS-UCNP-KR-LP in both buffer and cell culture medium including FBS, no significant changes were observed in the PL intensity and polydispersity index over a two-week period (Additional file 1: Figure S9).

### FRET efficiency between UCNPs and KR-LP

**Fig. 3:**
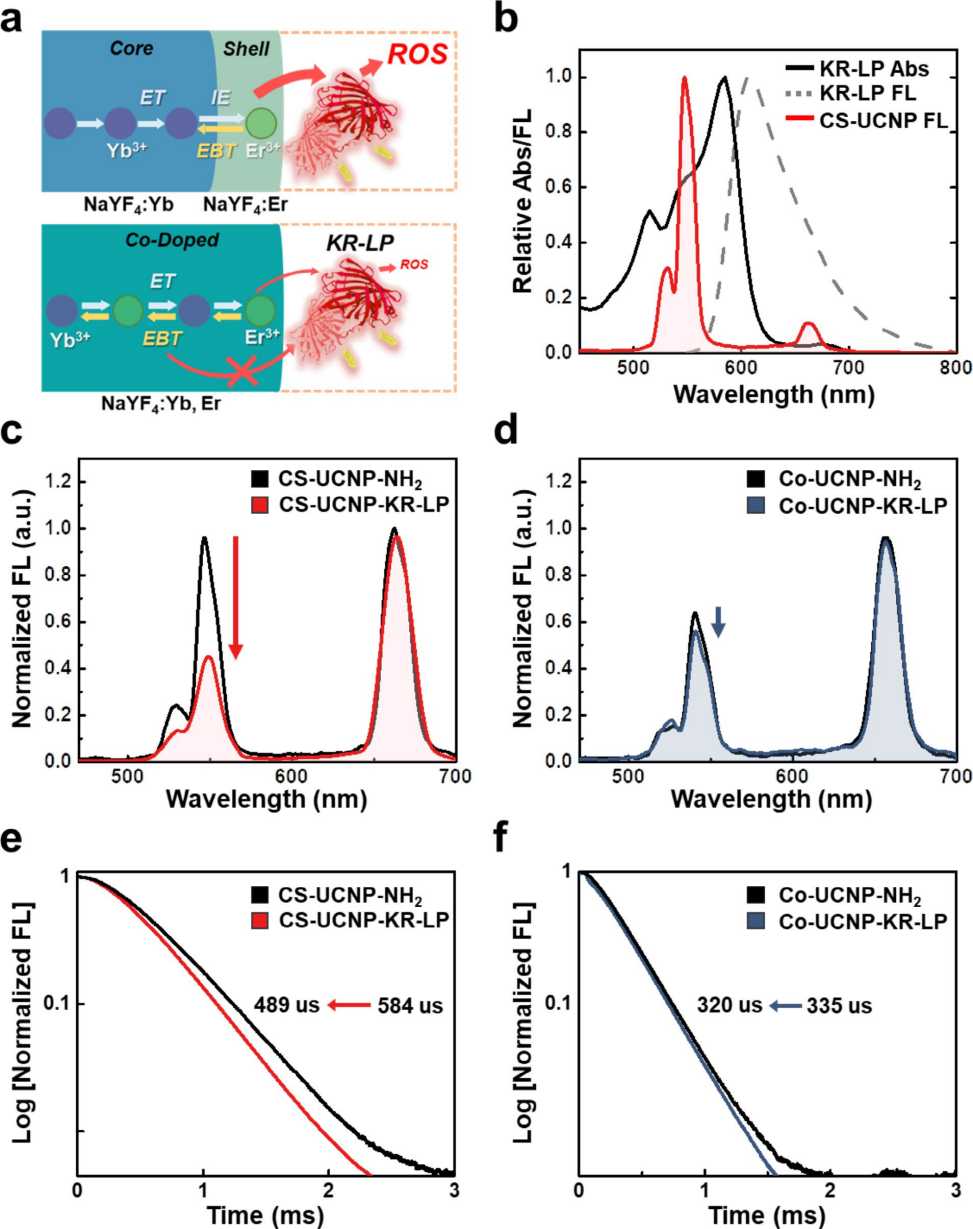
Measurement of FRET between UCNPs and KR-LP. **a** Schematic of ET pathway from CS-UCNPs (top) or Co-UCNPs (bottom) to KR-LP. **b** Spectral overlap between the emission of CS-UCNPs (donor) and the absorbance of KR-LP (acceptor). **c−d** Changes in the fluorescence (FL) intensity spectra of CS-UCNP-NH_2_ (**c**) and Co-UCNP-NH_2_ (**d**) in the absence and presence of KR-LP. **e−f** PL decay time curves of CS-UCNP-NH_2_ (**e**) and Co-UCNP-NH_2_ (**f**) in the absence and presence of KR-LP under 980 nm excitation and 550 nm emission. FRET, fluorescence resonance energy transfer

To investigate whether ET can occur between CS-UCNPs and KR in the nanocomposite, we examined FRET efficiency from CS-UCNPs as an energy donor to KR as an energy acceptor. For comparison, FRET efficiency between Co-UCNPs and KR was also measured to demonstrate the superior properties of CS-UCNPs compared with Co-UCNPs (Fig. 3). CS-UCNPs may allow efficient FRET in close proximity (< 10 nm) between the Er^3+^ concentrated in the shell and the surface-attached KR [35, 36]. On the other hand, Co-UCNPs may cause a reduction in FRET efficiency due to the energy loss by the strong EBT of Er^3+^ in the co-doped core given that Er^3+^ ions in Co-UCNPs are randomly distributed a few nanometers apart at low concentrations (Fig. 3a) [37]. There was a large overlap between the emission spectrum of CS-UCNPs (or Co-UCNPs) and the absorbance spectrum of KR-LP (Fig. 3b). In comparison with CS-UCNP-NH_2_, CS-UCNP-KR-LP showed a marked decrease (52%) in the 550 nm emission of donor NPs under 980 nm irradiation (Fig. 3c). However, compared with Co-UCNP-NH_2_, Co-UCNP-KR-LP showed a marginal decrease (18%) in the 550 nm emission of donor NPs (Fig. 3d). This intensity-based FRET was further corroborated by lifetime-based FRET measurements, which remain unaffected by initial emission intensity, nanoparticle concentration, and radiative photon reabsorption. Notably, the presence of KR-LP in each UCNP reduced the average PL decay time to different degrees, resulting in a relatively large decrease from 584 µs to 489 µs (for CS-UCNPs, Fig. 3e) and a relatively small decrease from 335 µs to 320 µs (for Co-UCNPs, Fig. 3f). These results indicated that nonradiative ET from CS-UCNPs to KR occurred more effectively than that from Co-UCNPs. Based on the PL decay time, the nonradiative ET efficiency of CS-UCNPs was 16.3% in the presence of an energy acceptor, which was 3.6-fold higher than that of Co-UCNPs (4.5%). Moreover, when controlling the concentration of Er^3+^ ion activators in CS-UCNPs (NaYF_4_:40% Yb^3+^@NaYF_4_:*x*% Er^3+^; *x* = 2, 4, 10 mol%), the most substantial reduction in the PL decay time was observed in the presence of KR-LP, specifically at a concentration of 2% Er^3+^ (Additional file 1: Figure S10). Therefore, the improved FRET efficiency of CS-UCNPs may be primarily attributed to the confined Er^3+^ in the outer layer with a short donor-acceptor distance, which is favorable for harvesting more excitation energy by the NaYF_4_:40% Yb^3+^ core [38].

### NIR-induced ROS generation of UCNP-KR-LP

**Fig. 4:**
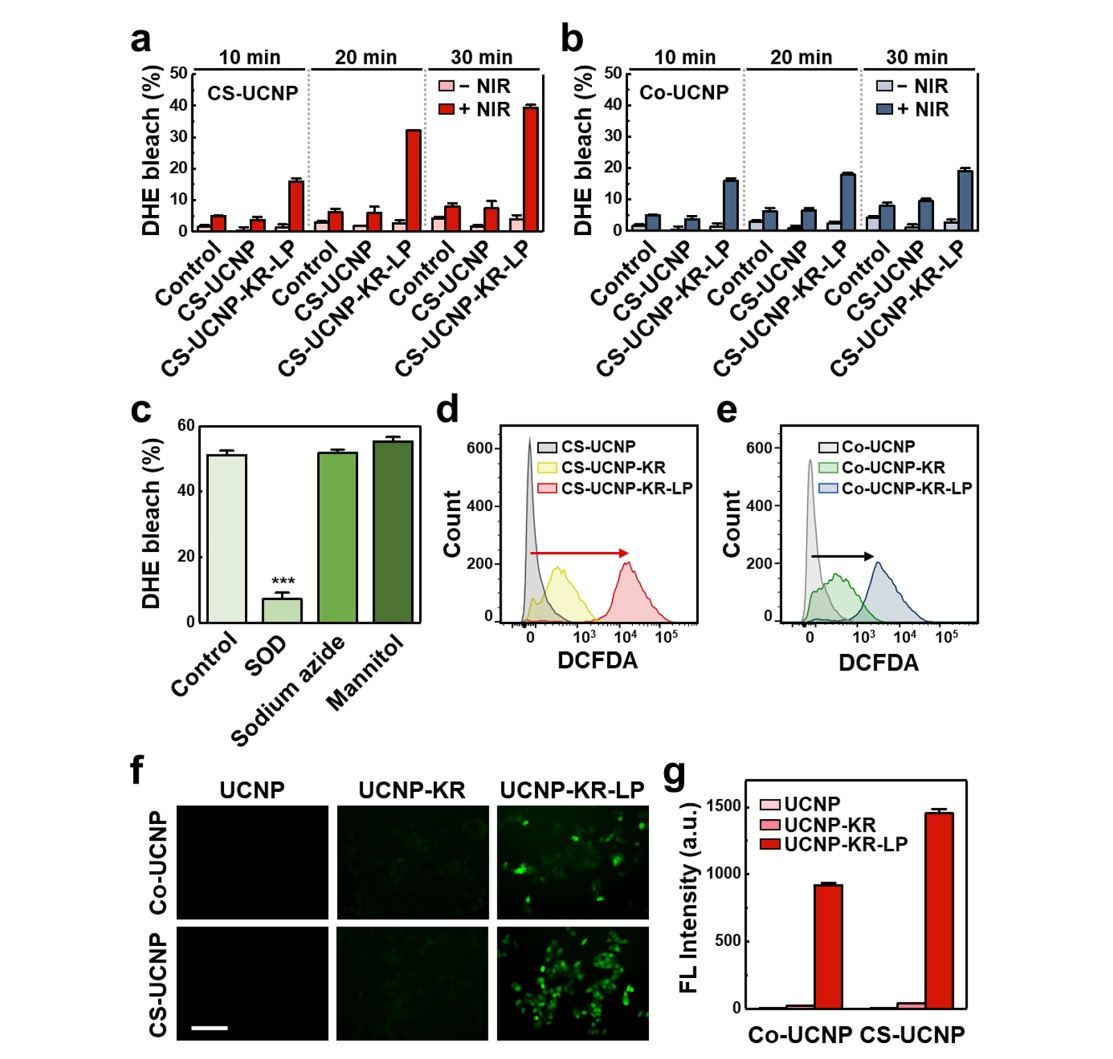
NIR-induced ROS generation of UCNP-KR-LP in cancer cells. **a−b** Detection of superoxide (O_2_^•−^) generation by CS-UCNPs (**a**) and Co-UCNPs (**b**) based on the DHE bleaching effect. The bleaching effects of the control (no NP), unmodified NP (CS-UCNP or Co-UCNP), and modified NP (CS-UCNP-KR-LP or Co-UCNP-KR-LP) groups were examined in the absence and presence of NIR irradiation over time (10−30 min). **c** Effect of different ROS scavengers on superoxide generation. CS-UCNP-KR-LP was treated with three ROS scavengers (SOD, sodium azide, and mannitol) after NIR irradiation for 30 min. The significant difference among scavengers was evaluated (***P < 0.001, *n* = 3, one-way ANOVA with post-hoc Tukey’s test). **d−e** Quantitative FACS analysis of intracellular ROS generation in DCFDA-responsive MCF-7 cell populations after treatment with unmodified or modified UCNPs (with KR or KR-LP). DCFDA was added to the cell culture medium before NIR irradiation at 980 nm. **f** Representative confocal images of intracellular ROS signals detected using DCFDA (green) in MCF-7 cells after treatment with unmodified or modified UCNPs (with KR or KR-LP). Experimental conditions were similar to those of FACS. White scale bar = 120 μm. **g** Quantitative FL analysis of DCFDA intensity from confocal images in three independent experiments. DHE, dihydroethidium; SOD, superoxide dismutase, FACS, fluorescence-activated cell sorting; DCFDA, 2ʹ,7ʹ-dichlorofluorescein diacetate

To investigate ROS generation by the two types of UCNP-KR-LP (i.e., Co-UCNP-KR-LP and CS-UCNP-KR-LP) under NIR irradiation, we measured ROS generated by the nanocomposites in vitro using dihydroethidium (DHE), a fluorogenic probe specific for superoxide radicals (O_2_^•−^) (Fig. 4a−c). KR is known to predominantly generate superoxide/ROS by photoactivation [22]. Superoxide inhibits the fluorescence of DHE, resulting in a bleaching effect. In comparison with control groups (no UCNPs; less than 5% as background) and UCNPs without KR-LP (only up to ~ 8%), UCNP-KR-LP exhibited greater DHE bleaching with increasing NIR irradiation over time (10−30 min); specifically, CS-UCNP-KR-LP (up to ~ 40%) showed greater DHE bleaching than Co-UCNP-KR-LP (up to ~ 18%) (Fig. 4a−b). In contrast, under conditions without NIR irradiation, no significant DHE bleaching was observed among all nanocomposites. Notably, the bleaching effect of CS-UCNP-KR-LP was inhibited only by superoxide dismutase (SOD; a superoxide scavenger) treatment in contrast to sodium azide (a single oxygen scavenger) and mannitol (a hydroxyl radical scavenger) treatments (Fig. 4c). These results strongly indicated that KR could generate superoxide by photoactivation, and NIR irradiation could allow CS-UCNP-KR-LP to generate more superoxide than that generated by Co-UCNP-KR-LP. In addition to in vitro ROS generation by UCNP-KR-LP under NIR irradiation, we further investigated the cell-specific ROS formation of UCNP-KR and UCNP-KR-LP in live cancer cells using fluorescence-activated cell sorting (FACS) and confocal fluorescence imaging (Fig. 4d−g). When 2ʹ,7ʹ-dichlorofluorescein diacetate (DCFDA) was used as a fluorogenic indicator for ROS formation in live cells, treatment of cultured MCF-7 cells with UCNP-KR-LP (CS-UCNP-KR-LP or Co-UCNP-KR-LP) induced the strong fluorescence signal of DCFDA after 30 min of NIR irradiation, in contrast to that following treatment with UCNP (CS-UCNP or Co-UCNP) and UCNP-KR (CS-UCNP-KR or Co-UCNP-KR). Based on FACS analyses (Fig. 4d−e) and confocal images (Fig. 4f−g), ROS generation by CS-UCNP-KR-LP was increased compared with that by Co-UCNP-KR-LP under NIR irradiation, which demonstrated the superior properties of CS-UCNPs with improved FRET efficiency. Conversely, without NIR irradiation, no fluorescence signal of DCFDA was observed in live cells treated with each nanocomposite (Additional file 1: Figure S11). These results imply that the two types of UCNP-KR-LP could selectively bind to cancer cells via ligand-receptor interactions, thus allowing NIR-induced ROS production in cancer cells. The findings also suggest that CS-UCNP-KR-LP may be more effective than Co-UCNP-KR-LP in inducing cell death.

### NIR-induced cytotoxic effect of UCNP-KR-LP on various cancer cell lines

**Fig. 5:**
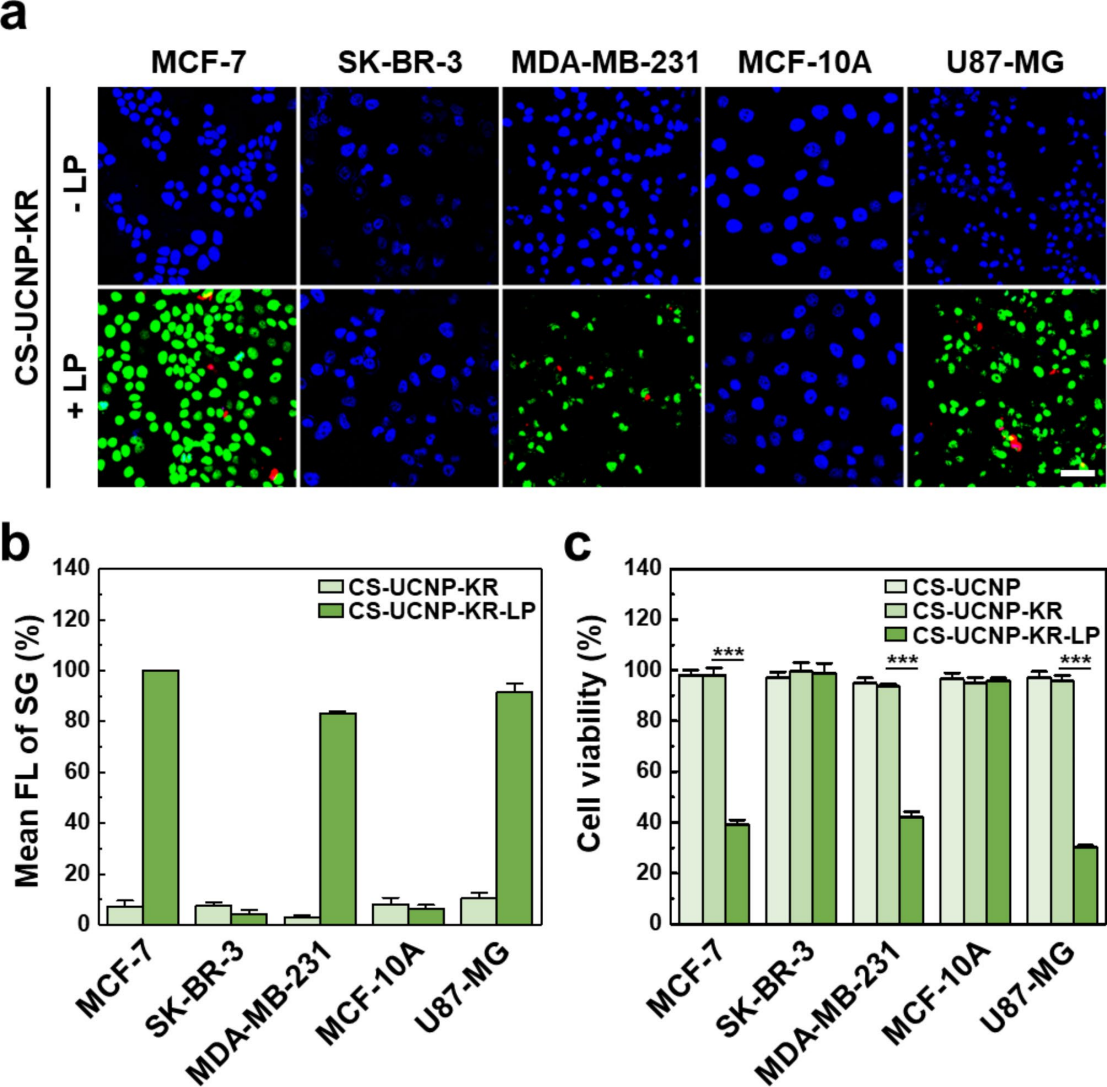
NIR-induced cytotoxic effect of UCNP-KR-LP on various cancer cell lines. **a** Representative confocal images of 5 cancer cell lines (MCF-7, SK-BR-3, MDA-MB-231, MCF-10 A, and U-87MG) with SG/DAPI double staining after treatment with CS-UCNP-KR (top) or CS-UCNP-KR-LP (bottom) followed by NIR irradiation at 980 nm for 30 min. Blue, green, and red indicate DAPI, SG, and KR, respectively. Scale bar = 50 μm. **b** Quantitative FL analysis of SG intensity from confocal images in three independent experiments. **c** MTT assay of the cell viability of 5 cancer cell lines. The cells were treated with unmodified or modified CS-UCNPs (every 200 µg/mL) followed by NIR irradiation at 980 nm (1 W/cm^2^ for 30 min). The significant difference in cell viability between CS-UCNP-KR and CS-UCNP-KR-LP was evaluated (***P < 0.001, *n* = 3, one-way ANOVA with post-hoc Tukey’s test). DAPI, 4′,6-diamidino-2-phenylindole; SG, SYTOX Green; MTT, 3-(4,5-dimethylthiazol-2-yl)-2,5-diphenyl tetrazolium bromide

Next, we examined whether CS-UCNP-KR-LP can induce the cell death of various cancer cell lines through ROS generation under NIR irradiation (Fig. 5). The SYTOX Green (SG) and DAPI dyes were used to stain dead and live cells, respectively. Unlike DCFDA that is permeable to both live and dead cells, SG is permeable only to dead cells and binds to nucleic acids to emit green fluorescence. Cell-permeable DAPI emits blue fluorescence only in the nucleus of live cells. SG/DAPI double staining was performed on 5 cancer cell lines after treatment with CS-UCNP-KR or CS-UCNP-KR-LP under 980 nm NIR irradiation for 30 min, which showed that CS-UCNP-KR did not cause cell death in all cancer cell lines (top images in Fig. 5a). In contrast, CS-UCNP-KR-LP caused a significant increase in SG-stained dead cells together with a substantial decrease in DAPI-stained viable cells among MCF-7, MDA-MB-231, and U87-MG but not SK-BR-3 and MCF-10 A cells (bottom images in Fig. 5a). In three CS-UCNP-KR-LP-reactive cell lines, the cell surface receptor binding of CS-UCNP-KR-LP was also observed based on the red fluorescence from KR. The cytotoxicity results obtained from confocal imaging (Fig. 5b) were consistent with those obtained from conventional cytotoxicity assay using chromogenic tetrazolium salts (Fig. 5c). In agreement with these results, the cytotoxic effect was increased not only with increasing concentration of UCNP composites (Additional file 1: Figure S12) but also with increasing NIR light irradiation time (Additional file 1: Figure S13). Among the various receptors expressed on the cancer cell surface, ITGB1 was previously found as an LP-binding receptor commonly expressed in the three cell lines [22, 28] but not in the SK-BR-3 cell line and non-tumorigenic MCF-10 A cell line with reduced ITGB1 expression. Although this observation suggests the target specificity of the LP via ITGB1, we cannot exclude the possibility that the LP may bind to other integrin subunits including α_5_β_1_, ανβ_3_, and ανβ_5_, which is similar to RGD (a representative tumor-homing peptide) [39, 40]. Importantly, the absence of either NIR light or CS-UCNPs did not result in nanocomposite binding or cell death in MCF-7 cells (Additional file 1: Figure S14); cancer cells were viable at different concentrations of CS-UCNPs in the absence of NIR light and at different power densities of the NIR laser in the absence of CS-UCNPs. The selective uptake of CS-UCNP-KR-LP by cancer cells, as compared to CS-UCNP or CS-UCNP-KR, was further validated through ICP-MS analysis (Additional file 1: Figure S15). These results indicated that CS-UCNP-KR-LP may specifically recognize cancer cells through ligand-receptor interactions and effectively damage target cancer cells under NIR irradiation.

### Skin tissue-penetrating PDT using NIR-irradiated UCNP-KR-LP

**Fig. 6:**
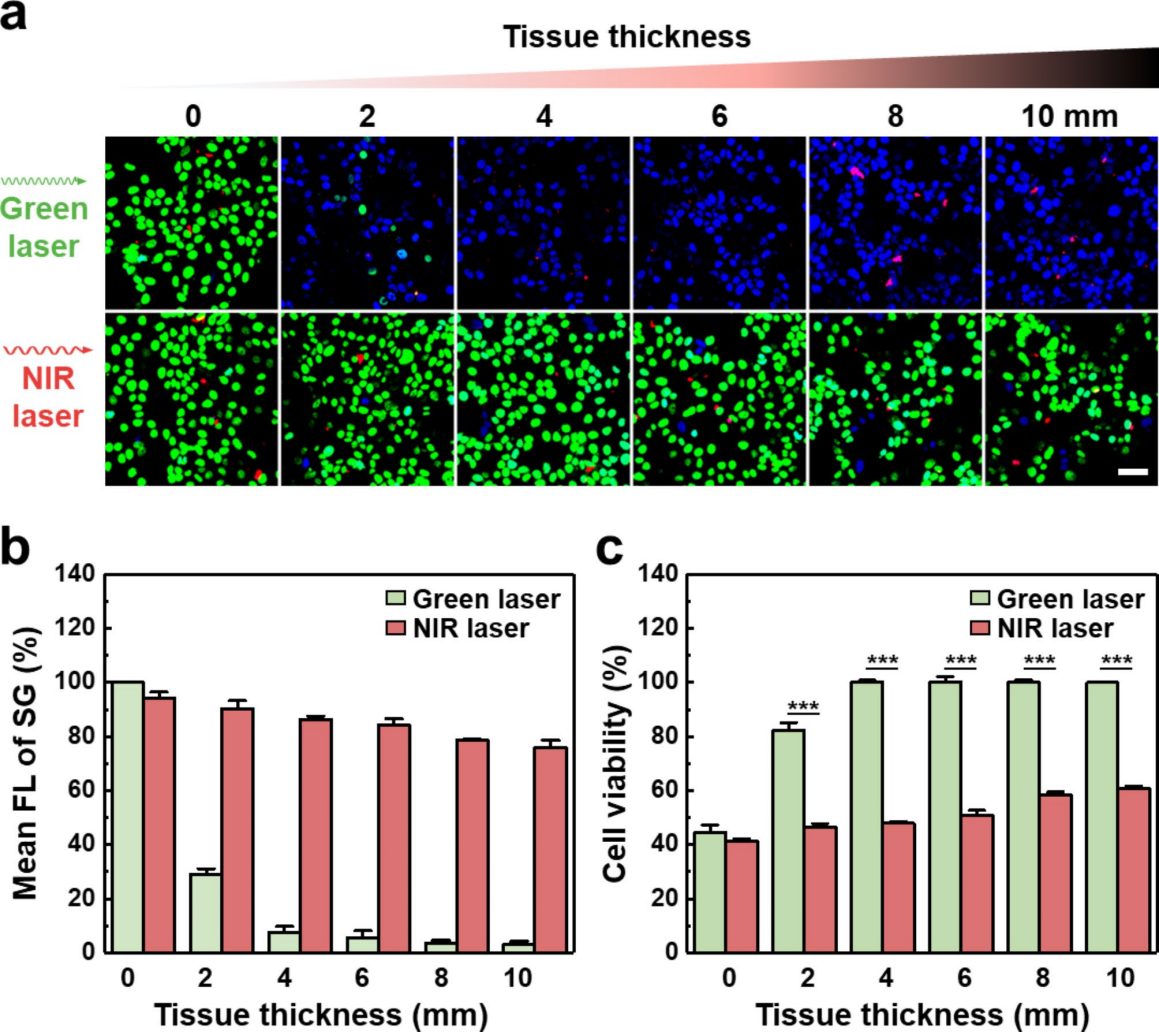
Skin tissue-penetrating PDT using NIR-irradiated UCNP-KR-LP. **a** Representative confocal images of live/dead MCF-7 cells with SG/DAPI double staining after treatment with CS-UCNP-KR-LP. The cells were cultured under porcine skin tissues of different thicknesses (0−10 mm), and the images were obtained after light irradiation with a green laser (0.5 W/cm^2^ at 550 nm; top) or a NIR laser (1 W/cm^2^ at 980 nm; bottom). Blue, green, and red indicate DAPI, SG, and KR, respectively. Scale bar = 50 μm. **b** Quantitative FL analysis of SG intensity from confocal images in three independent experiments under similar conditions. **c** MTT assay of cell viability according to tissue thickness. MCF-7 cells were incubated with CS-UCNP-KR-LP (200 µg/mL), followed by green or NIR irradiation for 30 min. Error bars indicate the standard deviations from triplicate experiments. The significant difference in cell viability between green and NIR irradiation was evaluated (***P < 0.001, *n* = 3, paired *t*-test with Shapiro-Wilk test)

To further assess the potential of CS-UCNP-KR-LP as a PDT agent, we investigated its NIR-responsive PDT ability to target cancer cells under biological barriers (Fig. 6). Porcine skin tissues of different thicknesses were used as a biological barrier under green or NIR light irradiation for 30 min (Additional file 1: Figure S16), and we examined the viability of MCF-7 cells located under the skin tissues by SG/DAPI staining after treatment with CS-UCNP-KR-LP. As shown in Fig. 6a−b, although green light irradiation at 550 nm induced significant cell death in the absence of porcine skin (i.e., 0 mm thickness) due to the direct excitation of KR without upconversion of ET, its cytotoxic effect was diminished with increasing tissue thickness increased; green light penetration was severely inhibited with thicknesses of more than 2 mm. This result is in agreement with the finding of a previous study showing that 1% of 550 nm light and 1% of 750 nm light reach a depth of around 3.0 and 5.4 mm, respectively [41]. In stark contrast, NIR light irradiation at 980 nm induced cell death with tissue thicknesses up to 10 mm. In the phototherapy of dermal abrasion, 980 nm light has been reported to be less effective than 810 nm light [42]. Therefore, in our study, the tissue penetration depth of NIR light was lower than that (~ 22 mm at 980 nm) of previously reported low-level laser therapy [43]. However, the cell death effect of our nanocomposite was greatly improved compared with that of previous conjugates of UCNPs and chemical PSs [44]. Given the findings, ROS generation and cell death should be considered in addition to the light transmission effect in tissue-penetrating PDT. Colorimetric 3-(4,5-dimethylthiazol-2-yl)-2,5-diphenyl tetrazolium bromide (MTT) assay of cells cultured under tissues of different thicknesses revealed that cell viability rates under green light irradiation were ~ 44% (0 mm), ~ 82% (2 mm), and ~ 100% (4 mm or more), whereas cell viability rates under NIR light irradiation were ~ 41% (0 mm) and ~ 60% (10 mm) (Fig. 6c). In contrast to ~ 100% cell death at 0 mm thickness in SG/DAPI-based imaging analysis, cell viability at 0 mm thickness was ~ 40% in MTT assay. Although this discrepancy in baseline cell viability may be attributed to factors such as reagent concentration, reaction time, and absorbance level in MTT assay, these results were consistent with those obtained from fluorescence imaging according to the skin tissue thickness. Therefore, NIR light may have higher tissue permeability than visible light, and that cancer cell death may be successfully achieved using CS-UCNP-KR-LP in deep-seated tissues.

### In vivo PDT effect of NIR-irradiated nanocomposites on tumor-xenograft mice

**Fig. 7:**
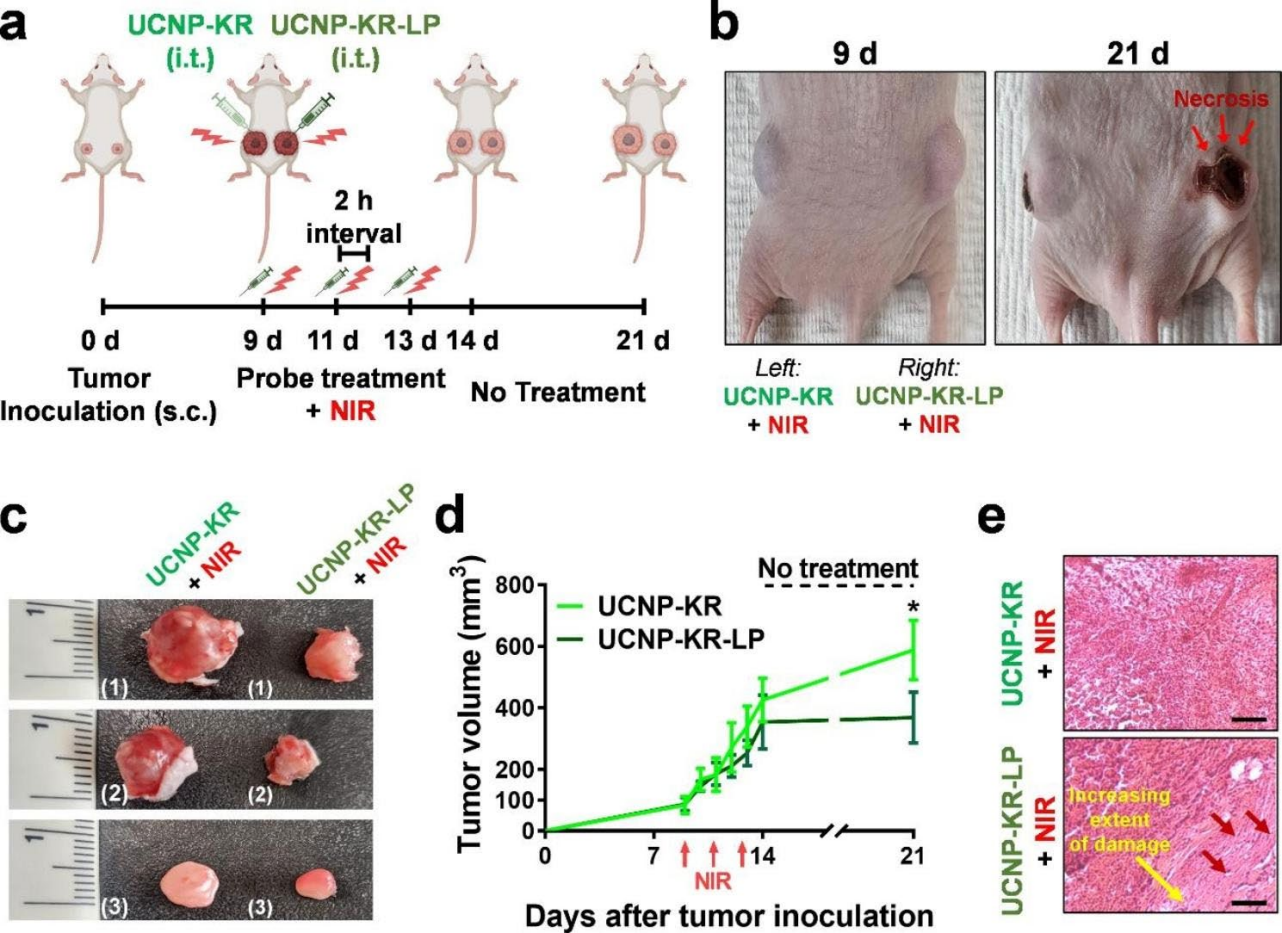
In vivo effect of nanocomposites in tumor xenograft mouse model. **a** Schematic illustration of experimental timeline. s.c., subcutaneous injection; i.t., intratumoral injection. Over the course of three repeated administration periods, a 10-min light irradiation with a NIR laser (1.5 W/cm^2^ at 980 nm) was applied after the intratumoral injection of either UCNP-KR or UCNP-KR-LP. **b** Representative images of nanocomposite/NIR-treated mice with bilateral tumors on day 9 (left image) and day 21 (right image). UCNP-KR and UCNP-KR-LP was administered to left and right flanks on the dorsal side of the mouse, respectively. **c** Three snapshot images of tumors treated with UCNP-KR (left) or UCNP-KR-LP (right) in BALB/c mice (#1−#3) on day 21. **d** Tumor growth curve in UCNP-KR or UCNP-KR-LP-treated BALB/c mice for 21 days after implantation. The error bars represent the standard deviation from triple mice. The significant difference in tumor volume between the groups treated with UCNP-KR (light green, 588 ± 167 mm^3^) or UCNP-KR-LP (dark green, 368 ± 143 mm^3^) on day 21 was evaluated (*P < 0.05, *n* = 3, paired *t*-test with Shapiro-Wilk test). **e** Representative H&E images of tumor regions treated with UCNP-KR (top) and UCNP-KR-LP (bottom) under the NIR irradiation condition. The eosin staining highlights the presence of marked coagulative necrosis, as indicated by the red arrows. Scale bar = 100 μm

To evaluate the in vivo PDT effect, two CS-UCNP composites (UCNP-KR and UCNP-KR-LP) were administered to MDA-MB-231-bearing BALB/c nude mice (Fig. 7). By applying three repetitive intratumoral injections of the nanocomposites and subsequent NIR irradiations to the tumor-growing sites in the mice (Fig. 7a−b), it was observed that the tumor regions treated with UCNP-KR-LP led to a more pronounced reduction in tumor growth compared to that treated with UCNP-KR (Fig. 7c−d). This finding indicates the elevated tumor specificity of the peptide ligand appended to the nanocomposite. Histological analysis of tissue sections from UCNP-KR-LP-treated tumors revealed an increased extent of damage (Fig. 7e). These results support the significant potential of LP-bearing CS-UCNP-KR for targeted therapy of tumors in vivo.

Based on the findings, our proteinaceous PS, in combination with CS-UCNPs, may offer distinct advantages over conventional chemical PSs or their conjugates with UCNPs. First, our PDT agent could allow excellent target specificity and water solubility with easy conjugation. As integrins serve as heterodimeric transmembrane receptors that mediate cell adhesion in cell-to-cell and cell-to-matrix interactions, some integrin subtypes including αvβ3, αvβ5, and α5β1 are known to be highly upregulated in metastatic tumor cells. For this reason, the peptide ligands for integrins have been utilized for targeted drug delivery and molecular imaging. Importantly, passive targeting, as performed with conventional PSs, is not adequate for in vivo PDT, wherase active targeting with cancer-targeting ligands can increase the local concentration of PSs in tumors and avoid side effects [45]. In particular, the LP used in this study is known to have better uptake efficiency by cancer cells compared with that of RGD [29]. Moreover, unlike chemical PSs or their nanoconjugates with high hydrophobicity, the recombinant protein in this study can be expressed with high solubility, which allows easy binding to UCNPs in solution with different combinations of ROS-generating proteins and peptide ligands. Notably, compared with chemical PSs, KR is less phototoxic in the dark and degades faster in vivo [21, 22], which can minimize photodamage to normal cells (e.g., MCF-10 A in our study). Second, our UCNP composite could contribute to high ROS production and a greater PDT effect in response to NIR irradiation. The high IET and minimal EBT of CS-UCNPs induced high FRET to KR under NIR irradiation, thus resulting in high ROS production and marked cell death in deep-seated tissues. Despite advances in long wavelength light-responsive PSs in the therapeutic window (700–1,100 nm) of biological tissues, chemical PSs allow limited light transmission to only ~ 3 mm below the skin due to significant light scattering and attenuation, thus requiring intense or prolonged light irradiation. In contrast, the cell death effect of our UCNP composite was more effective in deeper tissues (~ 10 mm), which is comparable to or better than the effect of NIR-excited PSs [46], two-photon excited NPs [47], or conventional UCNPs [48]. Considering that tissue depth-related effects depend on laser fluence (J/cm^2^), irradiance (W/cm^2^), or pulse structure, further research is needed to overcome in vivo depth limitations (several centimeters). Nevertheless, we anticipate that the designed nanocomposite would be advatageous for targeting deep-seated cancers with high on-target effects and low off-target effects.

## Conclusions

We developled photoprotein-bound UCNPs for the detection and treatment of deep-seated cancer cells to resolve the problem of specificity and solubility simultaneously. The chemically modified nanocomposite of CS-UCNPs with KR-LP (CS-UCNP-KR-LP) could induce rapid ROS formation and targeted cancer cell death under NIR irradiation through both efficient IET from NIR to green light and efficient FRET to KR. Notably, the introduction of the LP in the UCNP-protein conjugate allowed exclusive target specificity through peptide ligand-integrin receptor interactions on the cancer cell membrane, thus leading to targeted cell death in close proximity. In contrast, the cytotoxic effect was lower under LP- or NIR-deficient conditions. Most importantly, unlike visible green light irradiation, NIR irradiation empowered CS-UCNP-KR-LP to effectively eliminate cancer cells positioned deep beneath porcine skin tissues up to a depth of 10 mm, while also suppressing tumor growth in an in vivo mouse model. Owing to its high target specificity and sensitivity to NIR, our developed nanocomposite holds great promise as an innovative PDT agent for effectively treating a wide range of deep-seated tumors.

### Electronic supplementary material

Below is the link to the electronic supplementary material.

Supplementary Material 1: Additional file 1: Figure S1. Elemental analysis of CS-UCNPs by energy-dispersive X-ray spectroscopy. Figure S2. X-ray diffraction patterns of oleic acid-capped Co-UCNPs and CS-UCNPs. Figure S3. TEM image of Co-UCNPs. Figure S4. TEM images of Co-UCNPs and CS-UCNPs. Figure S5. TEM images of CS-UCNPs. Figure S6. Effect of Yb^3+^ concentratons on the ET efficiency in UCNPs. Figure S7. SDS-PAGE and fluorescent gel images of KR and KR-LP. Figure S8. FT-IR spectrum of KR-LP. Figure S9.In vitro stability of CS-UCNP-KR-LP over a two-week period using PL intensity and polydispersity index. Figure S10. Effect of Er^3+^ concentrations (2−10%) in CS-UCNP-NH_2_ on the PL decay time. Figure S11. Flow cytometric analysis of intracellular ROS generation using DCFDA with CS-UCNPs or Co-UCNPs. Figure S12. MTT assay of cell viability according to nanocomposite concentration for 5 cancer cell lines. Figure S13. MTT assay of cell viability according to irradiation time for 5 cancer cell lines. Figure S14. MTT assay of cell viability of MCF-7 cells without either NIR irradiation or nanocomposites. Figure S15. Measurement of cellular uptake of three different CS-UCNPs using ICP-MS in cancer cells. Figure S16. Experimental setup for evaluating the tissue-penetrating effect of NIR irradiation on CS-UCNP-KR-LP in MCF-7 cells.

## Data Availability

The raw data and processed data required to reproduce these findings are available from the corresponding author upon request.
