# Supplementary material for: Photosensitizing deep-seated cancer cells with photoprotein-conjugated upconversion nanoparticles
Source: J Nanobiotechnology. 2023 Aug 19;21:279. doi: 10.1186/s12951-023-02057-0 (PMC10439569; doi:10.1186/s12951-023-02057-0)
Supplement: Supplementary file 1 — Supplementary Material 1: Additional file 1: Figure S1. Elemental analysis of CS-UCNPs by energy-dispersive X-ray spectroscopy. Figure S2. X-ray diffraction patterns of oleic acid-capped Co-UCNPs and CS-UCNPs. Figure S3. TEM image of Co-UCNPs. Figure S4. TEM images of Co-UCNPs and CS-UCNPs. Figure S5. TEM images of CS-UCNPs. Figure S6. Effect of Yb3+ concentratons on the ET efficiency in UCNPs. Figure S7. SDS-PAGE and fluorescent gel images of KR and KR-LP. Figure S8. FT-IR spectrum of KR-LP. Figure S9.In vitro stability of CS-UCNP-KR-LP over a two-week period using PL intensity and polydispersity index. Figure S10. Effect of Er3+ concentrations (2−10%) in CS-UCNP-NH2 on the PL decay time. Figure S11. Flow cytometric analysis of intracellular ROS generation using DCFDA with CS-UCNPs or Co-UCNPs. Figure S12. MTT assay of cell viability according to nanocomposite concentration for 5 cancer cell lines. Figure S13. MTT assay of cell viability according to irradiation time for 5 cancer cell lines. Figure S14. MTT assay of cell viability of MCF-7 cells without either NIR irradiation or nanocomposites. Figure S15. Measurement of cellular uptake of three different CS-UCNPs using ICP-MS in cancer cells. Figure S16. Experimental setup for evaluating the tissue-penetrating effect of NIR irradiation on CS-UCNP-KR-LP in MCF-7 cells. [file 12951_2023_2057_MOESM1_ESM.pdf]

Supplementary Information

Photosensitizing deep-seated cancer cells with photoprotein-conjugated upconversion nanoparticles

Sung Hyun Park†, Soohyun Han†, Sangwoo Park†, Hyung Shik Kim, Kyung-Min Kim, Suyeon Kim, Dong Yun Lee*, Joonseok Lee*, and Young-Pil Kim*

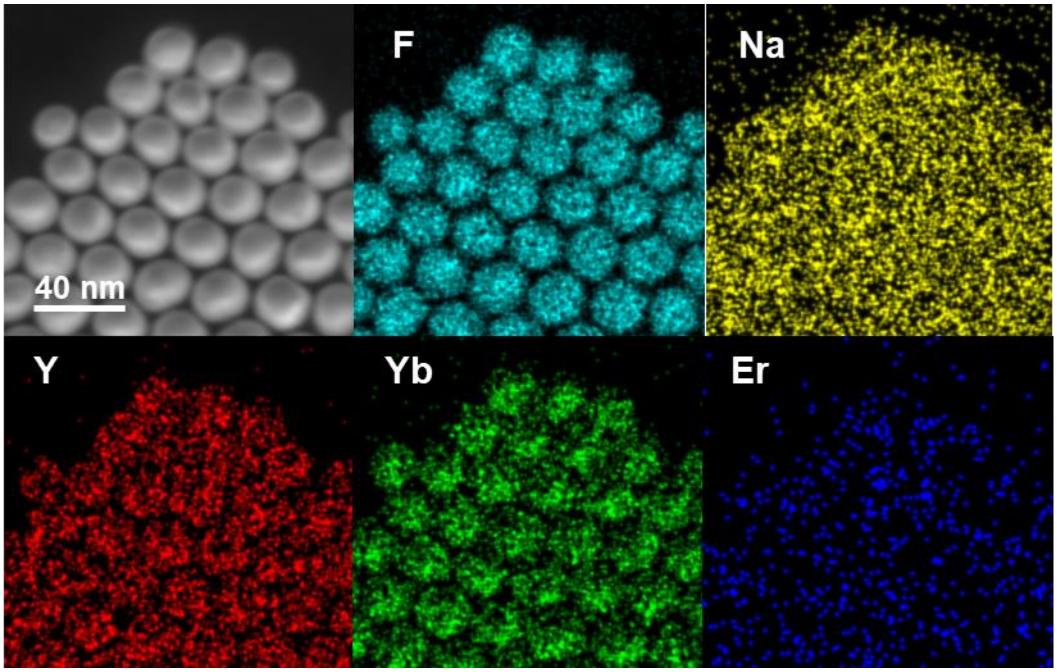

Figure S1. Elemental characteristics of CS-UCNPs ($NaYF_4$:Yb^{3+}@$NaYF_4$:Er^{3+}) determined by energy-dispersive X-ray spectroscopy (EDS).

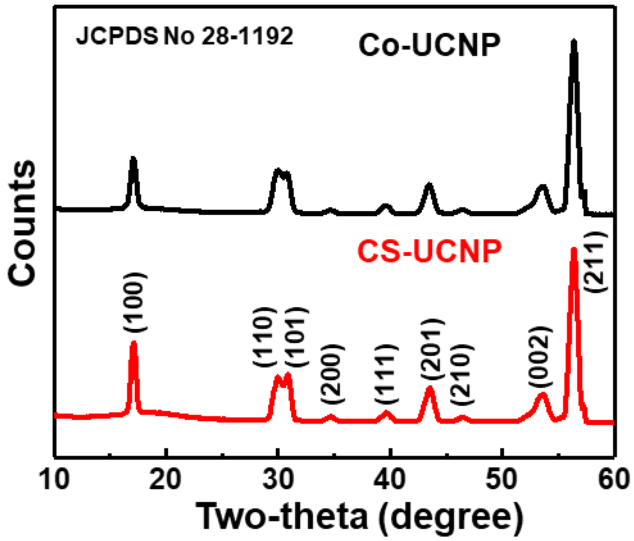

Figure S2. X-ray diffraction (XRD) patterns of oleic acid (OA)-capped co-doped (Co)-UCNPs ($NaYF_4$:20 % Yb^{3+}, 2% Er^{3+}) and core-shell (CS)-UCNPs ($NaYF_4$:40% Yb^{3+}@$NaYF_4$:2% Er^{3+}). The peaks of CS-UCNPs were labeled based on the standard hexagonal $NaYF_4$ structure (JCPDS no. 28-1192).

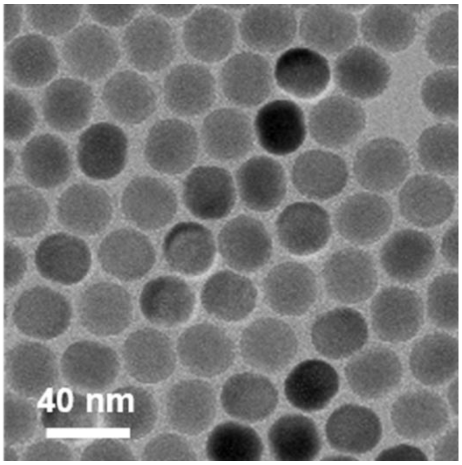

Figure S3. TEM image of Co-UCNPs ($NaYF_4$:20% Yb^{3+}, 2% Er^{3+}). The mean diameter of Co-UCNPs was maintained at ~27 nm.

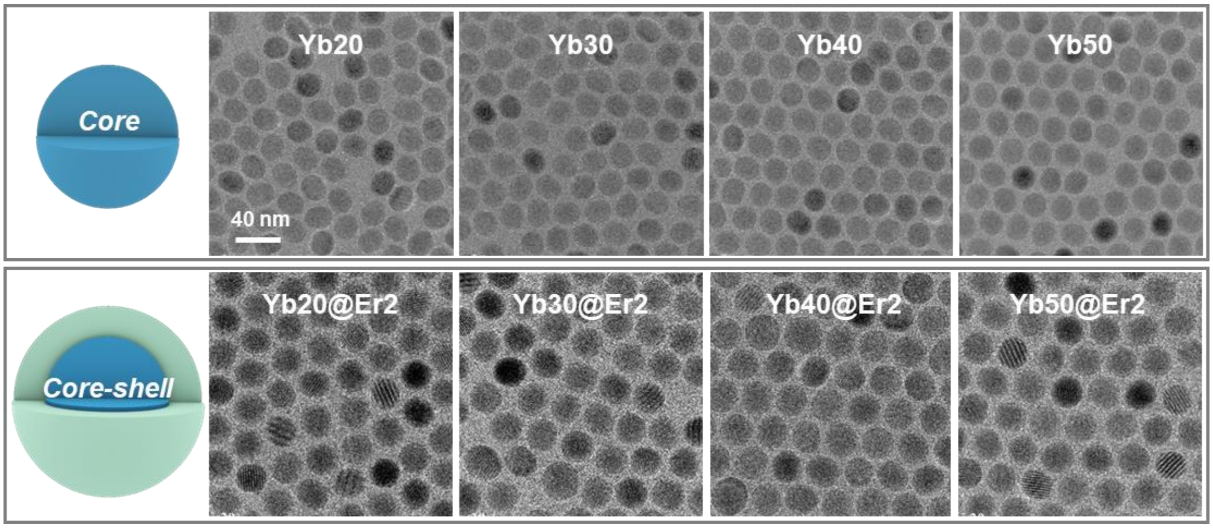

Figure S4. TEM images of Co-UCNPs ($NaYF_4$:*x*% Yb^{3+}; *x* = 20, 30, 40, 50 mol%) and CS-UCNPs ($NaYF_4$:*x*% Yb^{3+}@$NaYF_4$:2% Er^{3+}; *x* = 20, 30, 40, 50 mol%). The mean diameters of Co-UCNPs and CS-UCNPs were maintained at ~18 nm and ~27 nm, respectively.

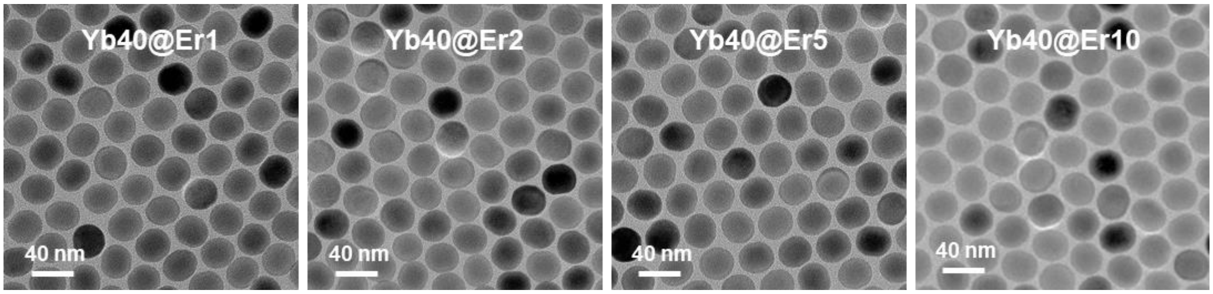

Figure S5. TEM images of CS-UCNPs ($NaYF_4$:40% Yb^{3+}@$NaYF_4$:*y*% Er^{3+}; *y* = 1, 2, 5, 10 mol%). The diameters were maintained at ~27 nm.

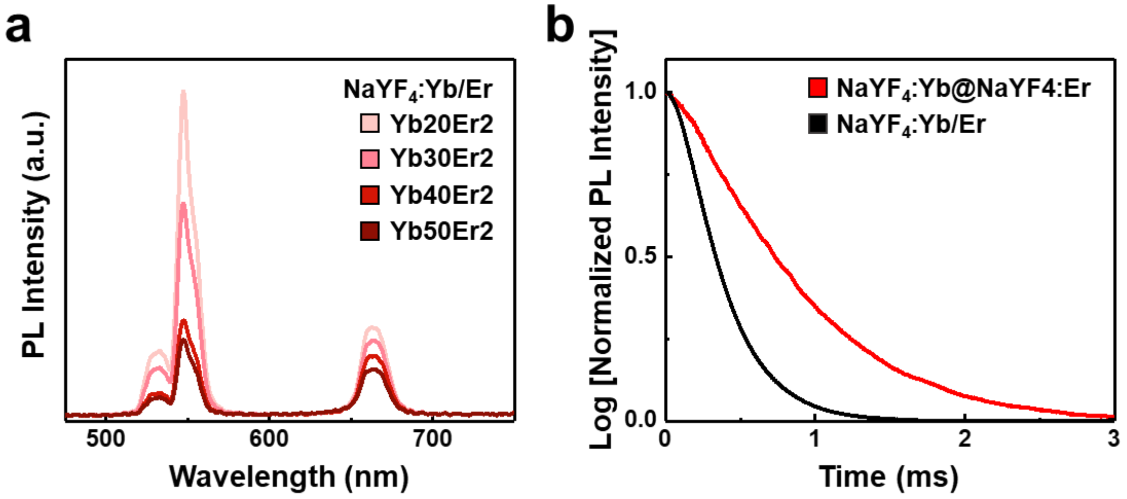

Figure S6. Effect of Yb^{3+} concentratons on the ET efficiency in UCNPs. **a** Photoluminescence (PL) spectra of Co-UCNPs ($NaYF_4$:*x*% Yb^{3+}, 2% Er^{3+}; *x*=20, 30, 40, 50 mol%) at upconversion emissions according to Yb^{3+} concentration. **b** PL decay time curves of CS-UCNPs ($NaYF_4$:40% Yb^{3+}@$NaYF_4$:2% Er^{3+}, red line) and Co-UCNPs ($NaYF_4$:20% Yb^{3+}, 2% Er^{3+}, black line). The Er^{3+} emission was measured at 550 nm.

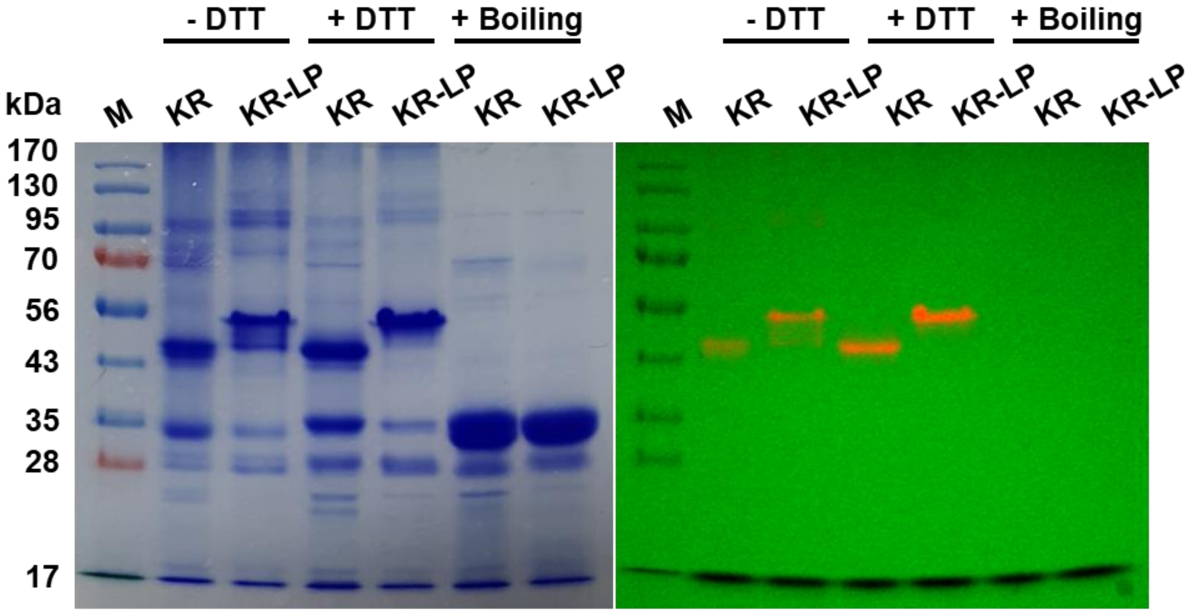

Figure S7. SDS-PAGE (left) and fluorescent gel (right) images of KR and KR-LP. 12% SDS-PAGE was run under non-reducing (−DTT), reducing (+DTT), or denaturing (boiling) conditions. A fluorescence image was obtained using a custom-built transilluminator equipped with a LED (at an excitation wavelength of 540−565 nm) and an emission filter (at an emission wavelength of 520−620 nm). The molecular weights of KR and KR-LP based on the amino acid sequence were 26.3 kDa and 27.6 kDa, respectively (their dimers were 52.6 kDa and 55.2 kDa, respectively). M, molecular size marker; DTT, 1,4-dithiothreitol.

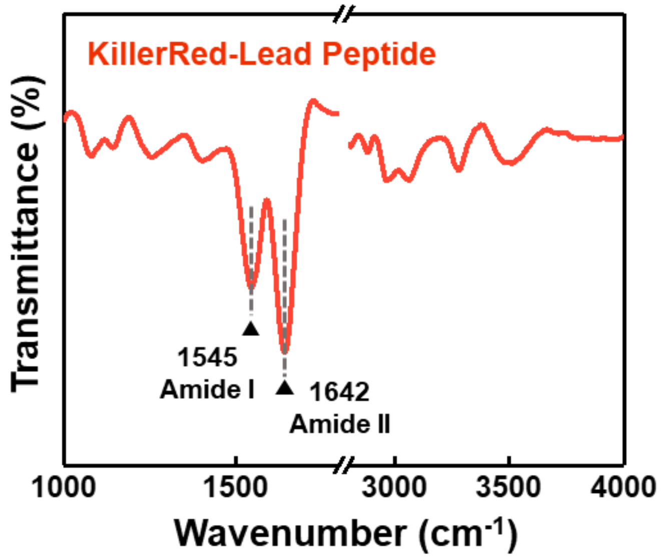

Figure S8. Fourier transform infrared spectroscopy (FT-IR) spectrum of KR-LP.

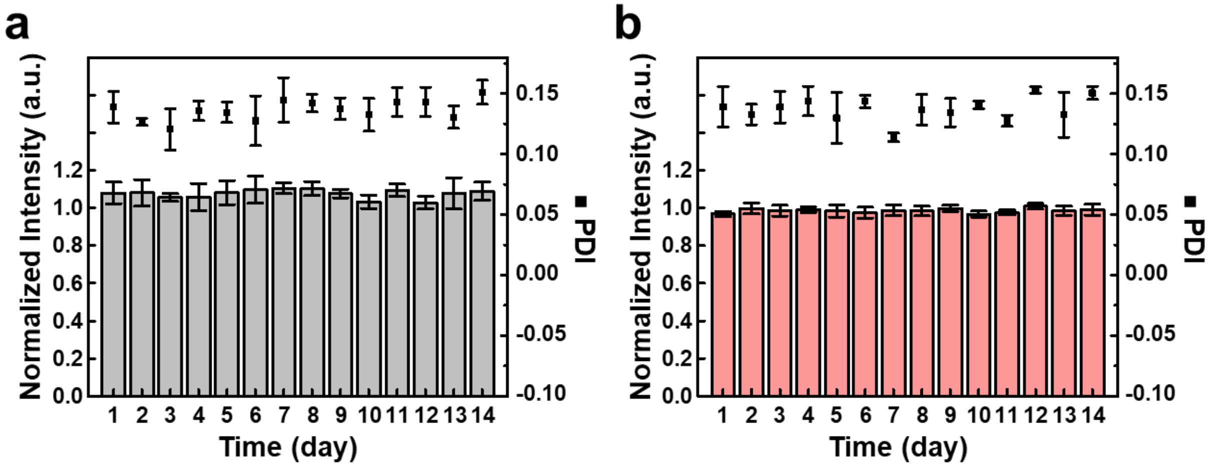

Figure S9. *In vitro* **s**tability of CS-UCNP-KR-LP over a two-week period using PL intensity and polydispersity index (PDI). **a** Stability of CS-UCNP-LP (final 0.5 mg/mL) in a 1× PBS solution. **b** Stability of CS-UCNP-LP (final 0.5 mg/mL) in DMEM cell culture medium supplemented with 10% FBS and 1% penicillin-streptomycin. The PL intensity was measured at an emission wavelength of 550 nm under 980 nm irradiation (1 W/cm^2), and PDI was monitored using dynamic light scattering.

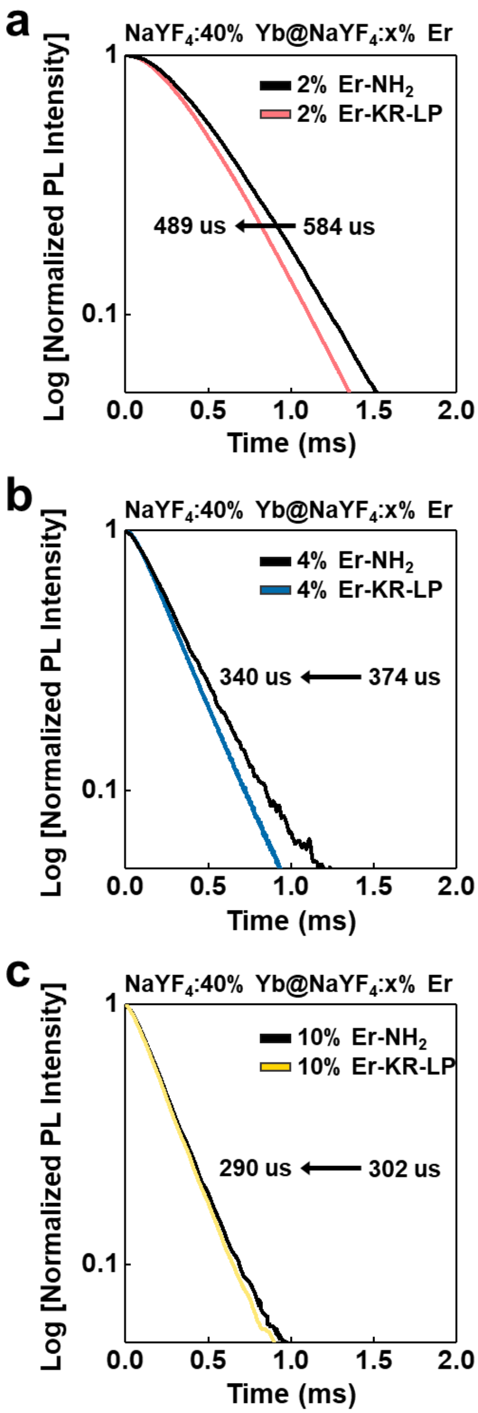

Figure S10. a-c Effect of Er^{3+} concentrations (2–10%) on the PL decay time curves of CS-UCNPs-NH_2 ($NaYF_4$:40% Yb^{3+}@$NaYF_4$:x% Er^{3+}; x = 2, 4, 10 mol%) in the absence and presence of KR-LP under 980 nm excitation and 550 nm emission.

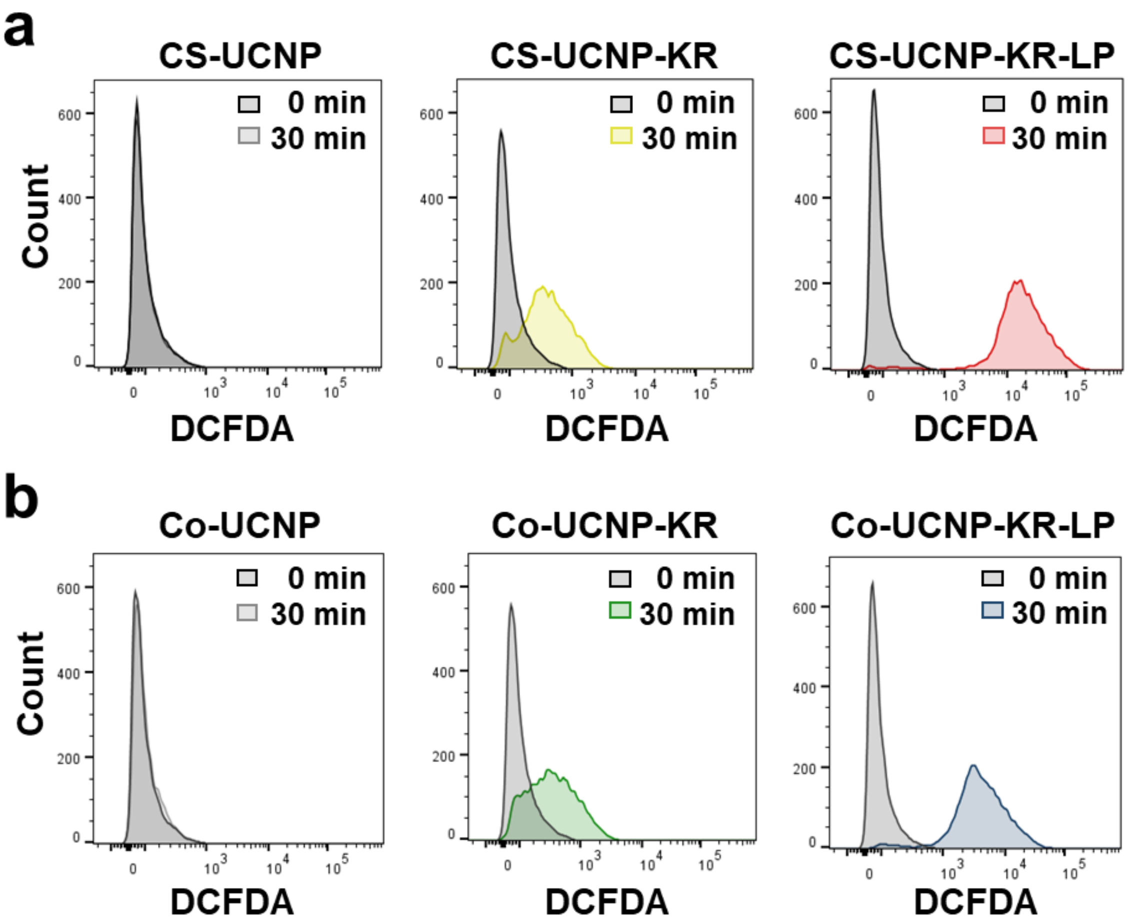

Figure S11. Flow cytometric analysis of intracellular ROS generation using DCFDA with **a** CS-UCNPs or **b** Co-UCNPs. MCF-7 cells were incubated with unmodified NPs (CS-UCNP or Co-UCNP) or modified NPs (CS-UCNP-KR, CS-UCNP-KR-LP, Co-UCNP-KR, and Co-UCNP-KR-LP), followed by further treatment with DCFDA. DCFDA-responsive cell population changes were quantitatively compared in each treatment group before (0 min) and after (30 min) 980 nm NIR irradiation.

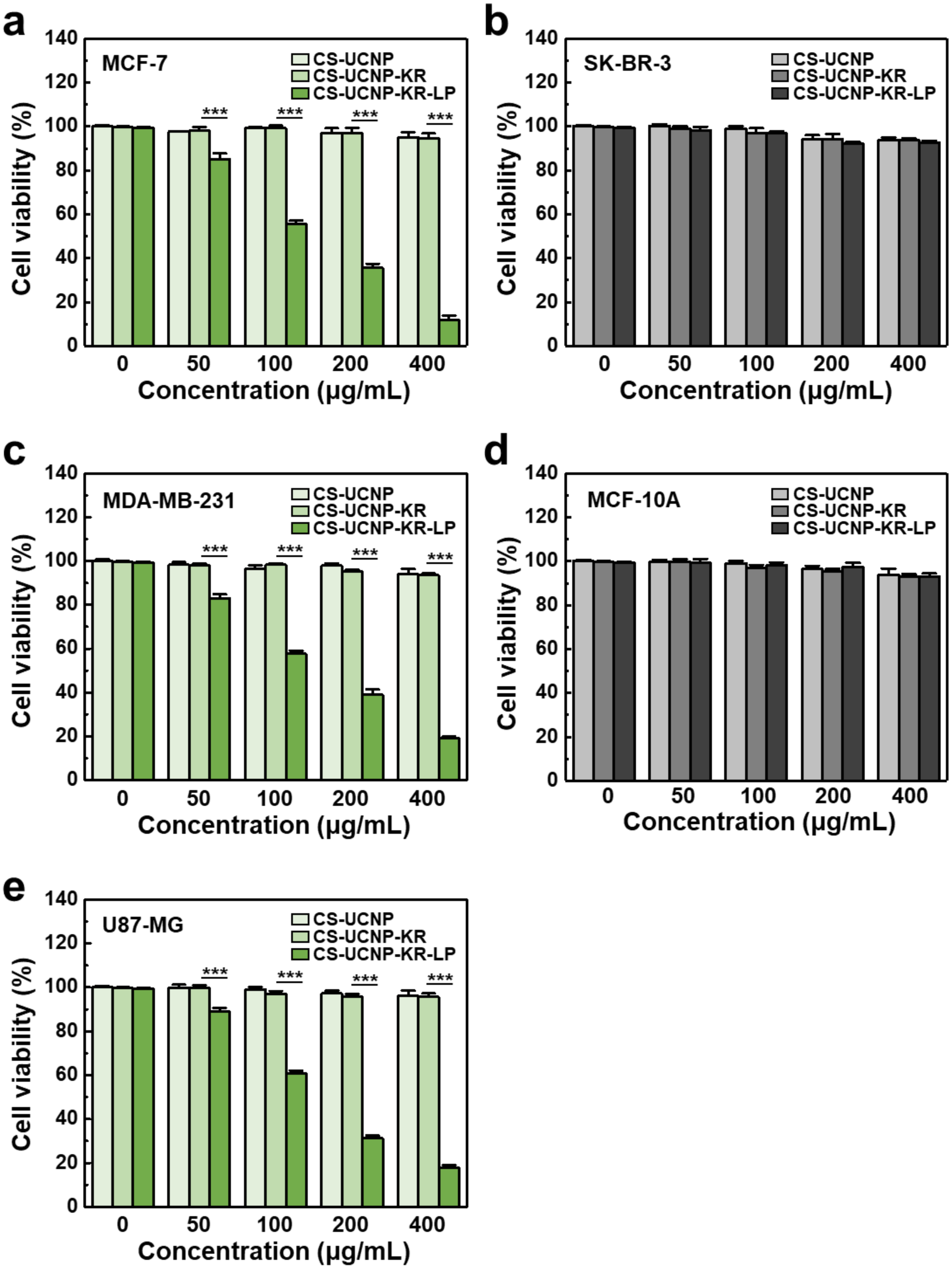

Figure S12. MTT assay of cell viability according to nanocomposite concentration (0, 50, 100, 200, and 400 μg/mL) for 5 cancer cell lines. **a** MCF-7, **b** SK-BR-3, **c** MDA-MB-231, **d** MCF-10A,

and **e** U87-MG cells. Three different nanocomposites (CS-UCNP, CS-UCNP-KR, or CS-UCNP-KR-LP) incubated with the cells were irradiated with NIR light at an intensity of 1 W/cm^2 for 30 min. Error bars indicate the standard deviations from triplicate experiments. The significant difference in cell viability between CS-UCNP-KR and CS-UCNP-KR-LP was evaluated (***$P < 0.001$, n=3, one-way ANOVA with post-hoc Tukey's test).

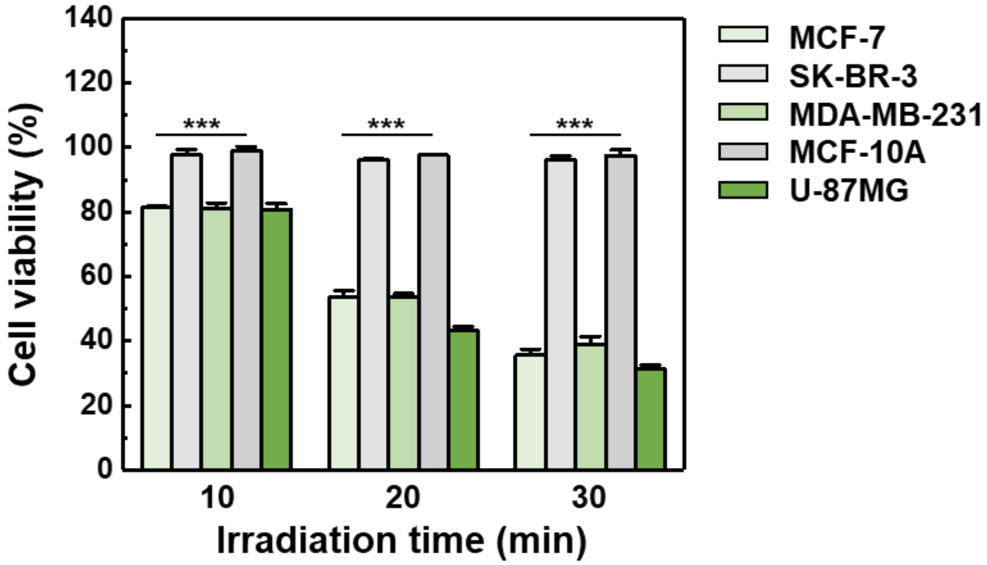

Figure S13. MTT assay of cell viability according to irradiation time (10, 20, and 30 min) for 5 cancer cell lines (MCF-7, SK-BR-3, MDA-MB-231, MCF-10A, and U87-MG). The cells were incubated with CS-UCNP-KR-LP (200 µg/mL), followed by NIR irradiation at an intensity of 1 W/cm^2. Error bars indicate the standard deviations from triplicate experiments. The significant difference in cell viability between MCF-7 and MCF-10A was evaluated (***$P < 0.001$, n=3, one-way ANOVA with post-hoc Tukey's test).

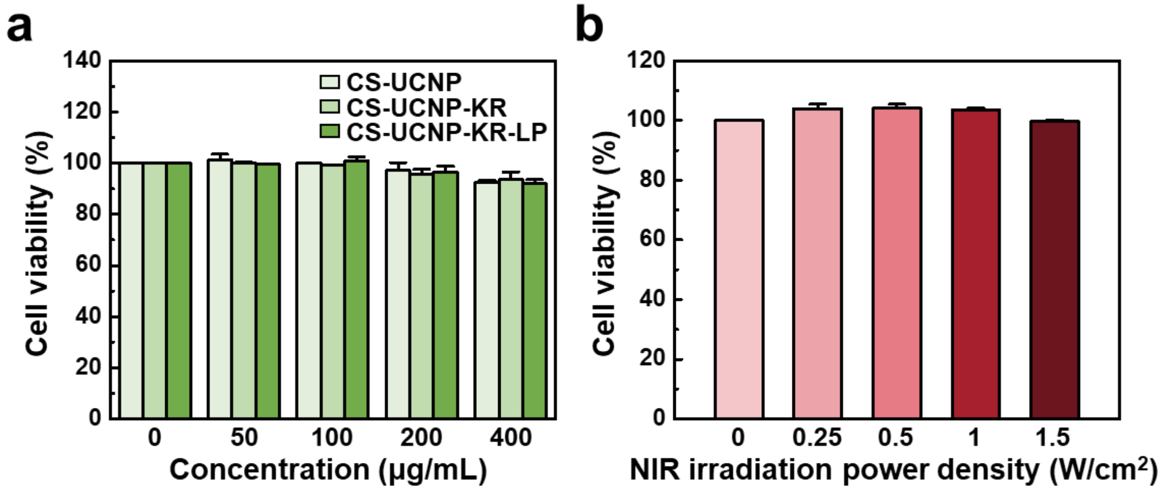

Figure S14. MTT assay of cell viability of MCF-7 cells without either **a** NIR irradiation or **b** nanocomposites. In graph (**a**), the cells were incubated with CS-UCNP, CS-UCNP-KR, or CS-UCNP-KR-LP at various concentrations (0–400 μg/mL) for 48 h without light irradiation. In graph (**b**), MCF-7 cells without nanocomposite treatment were irradiated for 30 min with different NIR laser power densities (0–1.5 W/cm^2). Error bars indicate the standard deviations from triplicate experiments.

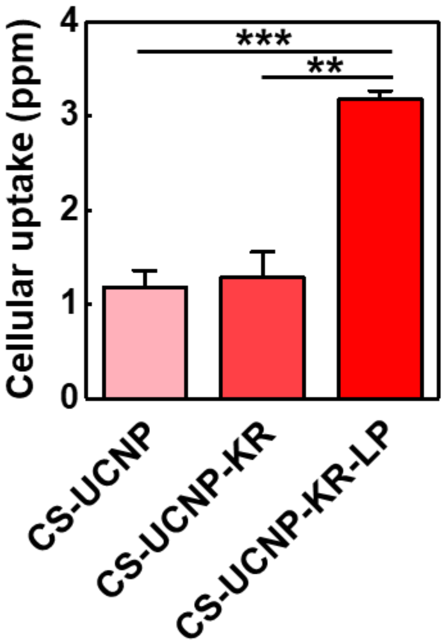

Figure S15. Measurement of cellular uptake of three different CS-UCNPs using ICP-MS in cancer cells. After MDA-MB-231 cells were treated with CS-UCNP, CS-UCNP-KR, or CS-UCNP-KR-LP (each 200 μg/mL) at 37 °C for 20 min, the cellular uptake of yttrium ions was quantified. The data are presented as mean ± SD (n=3). Significant difference between groups treated with different CS-UCNPs are statistically determined using a two-tailed unpaired t-test (**$P < 0.01$, ***$P < 0.001$).

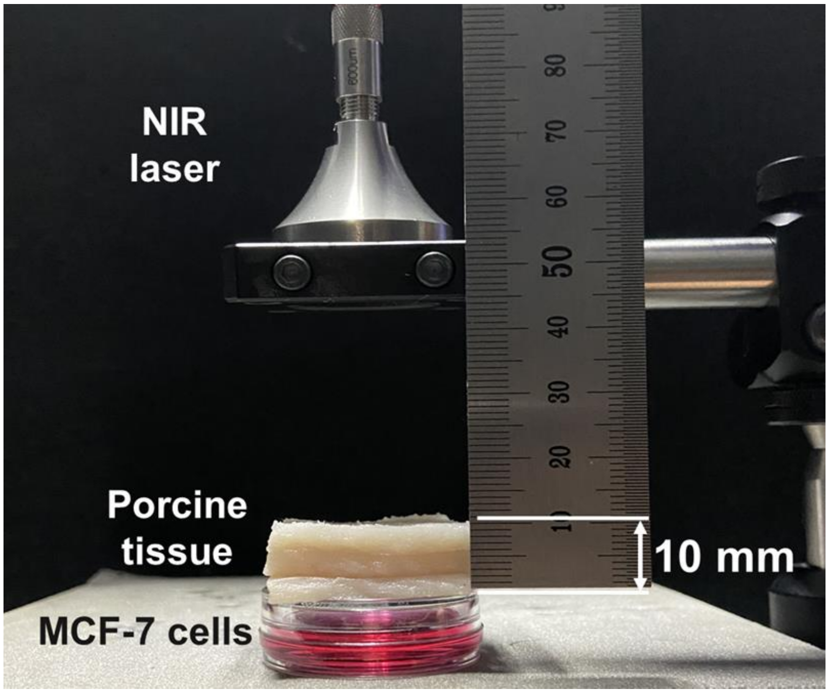

Figure S16. Experimental setup for evaluating the tissue-penetrating effect of NIR irradiation on CS-UCNP-KR-LP in MCF-7 cells. The cells were cultured under porcine skin tissues of different thicknesses (0−10 mm).
